# Screen‐Printing Technology for Scale Manufacturing of Perovskite Solar Cells

**DOI:** 10.1002/advs.202303992

**Published:** 2023-08-04

**Authors:** Changshun Chen, Chenxin Ran, Qing Yao, Jinpei Wang, Chunyu Guo, Lei Gu, Huchen Han, Xiaobo Wang, Lingfeng Chao, Yingdong Xia, Yonghua Chen

**Affiliations:** ^1^ Frontiers Science Center for Flexible Electronics Xi'an Institute of Flexible Electronics (IFE) Northwestern Polytechnical University Xi'an 710072 P. R. China; ^2^ Key Laboratory of Flexible Electronics (KLOFE) and Institution of Advanced Materials (IAM) School of Flexible Electronics (Future Technologies) Nanjing Tech University (NanjingTech) Nanjing Jiangsu 211816 P. R. China

**Keywords:** screen‐printing, perovskite solar cells, large‐scale fabrication, low‐cost technology

## Abstract

As a key contender in the field of photovoltaics, third‐generation thin‐film perovskite solar cells (PSCs) have gained significant research and investment interest due to their superior power conversion efficiency (PCE) and great potential for large‐scale production. For commercialization consideration, low‐cost and scalable fabrication is of primary importance for PSCs, and the development of the applicable film‐forming techniques that meet the above requirements plays a key role. Currently, large‐area perovskite films are mainly produced by printing techniques, such as slot‐die coating, inkjet printing, blade coating, and screen‐printing. Among these techniques, screen printing offers a high degree of functional layer compatibility, pattern design flexibility, and large‐scale ability, showing great promise. In this work, the advanced progress on applying screen‐printing technology in fabricating PSCs from technique fundamentals to practical applications is presented. The fundamentals of screen‐printing technique are introduced and the state‐of‐the‐art studies on screen‐printing different functional layers in PSCs and the control strategies to realize fully screen‐printed PSCs are summarized. Moreover, the current challenges and opportunities faced by screen‐printed perovskite devices are discussed. This work highlights the critical significance of high throughput screen‐printing technology in accelerating the commercialization course of PSCs products.

## Graphical abstract

Screen printing offers a high degree of functional layer compatibility, pattern design flexibility, and large‐scale ability, showing great promise. In this work, the advanced progress is presented on applying screen‐printing technology in fabricating perovskite solar cells (PSCs) from technique fundamentals to practical applications. This work highlights the critical significance of high throughput screen‐printing technology in accelerating the commercialization course of PSCs products.

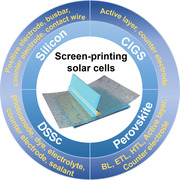

## 1 Introduction

The sustainable development of human society requires a stable and reliable energy supply, however, the depletion of traditional fossil fuels and associated environmental concerns have captured significant public attention. Therefore, incessantly exploring alternative, clean, and renewable sources of energy is urgent to bolster future sustainable development of human society, which not only enhances environmental quality but also fosters the advancement of global sustainability. Solar energy is a plentiful and eco‐friendly natural energy resource that has the significant potential to contribute to reducing the negative effects of greenhouse gases and achieving global carbon neutrality in the near future.^[^ ^1^, ^2^, ^3^, ^4^, ^5^, ^6^, ^7^, ^8^ ^]^ The earth could receive 1.49 × 10^22^ J solar energy every day, and utilization of only 1% of the energy from it can meet current global energy demands.^[^ ^9^ ^]^ Therefore, the development of efficient solar cell devices to convert solar energy into electrical energy plays a key role in energy saving and carbon emission reduction to solve the energy problem faced by mankind.

Currently, solar cells based on silicon material dominate the photovoltaic market over the past few decades, and the record PCE of silicon‐based solar cells has recently reached 26.8%.^[^ ^10^ ^]^ However, due to the considerably high manufacturing cost of silicon‐based solar cell products, the photovoltaic industry shows weak competitiveness, and only 2% of the world's energy supply is currently provided by solar energy.^[^ ^11^, ^12^, ^13^, ^14^ ^]^ To increase the contribution of photovoltaics to the global energy market, it is essential to reduce the cost and improve the PCE of unit solar cell devices.^[^ ^15^, ^16^, ^17^ ^]^

In recent years, solar cells based on organic‐inorganic hybrid perovskite materials have emerged as promising third‐generation photovoltaic technology due to their low material cost and excellent photovoltaic performance.^[^ ^18^ ^]^ Most importantly, perovskite material is able to be processed by solution‐based printing or coating technologies, which further reduces the manufacturing cost of PSCs, especially for large‐scale commercialization. Therefore, these advances in PSCs lead to extensive research on their potential as a viable alternative to conventional solar cells for large‐scale and high‐throughput manufacturing.^[^ ^9^, ^19^, ^20^, ^21^, ^22^, ^23^, ^24^, ^25^, ^26^ ^]^ Nevertheless, the fabrication of efficient and stable PSCs at large‐area is still challenging, which hinders the development of PSCs. To produce large‐area PSCs, the key is the deposition of high‐quality perovskite film on large‐area substrate. However, the classical spin‐coating method used in lab‐scale film formation cannot meet the requirement, which is due to the inhomogeneous linear velocity at the different positions of the spinning substrate, making it impossible to produce homogeneous high‐quality perovskite film on a large‐area substrate. Therefore, there is a pressing need to develop simple, convenient, and efficient processing techniques for large‐area perovskite film fabrication to promote the large‐scale commercialization process of PSCs.

Currently, various available printing and coating technologies, such as blade coating, slot‐die coating, D‐bar coating, inkjet printing, spray printing, roll‐to‐roll printing, and screen‐printing, have been employed to deposit large‐area perovskite film for manufacturing scaled PSCs and their modules.^[^ ^27^, ^28^, ^29^, ^30^, ^31^ ^]^ For perovskite devices, almost all functional layer films can be prepared by the above printing method, including hole blocking layer, an electron‐transport layer, an insulating layer, a hole‐transport layer, a perovskite layer, counter electrode. These methods can be mainly classified into two types: conventional coating to color a substrate for aesthetic purposes and functional printing aiming at endowing the substrate with specific functionality, such as electrical conductivity or optical sensing.^[^ ^32^, ^33^, ^34^ ^]^ Among the available printing techniques, screen‐printing is widely regarded as one of the most prevalent technologies in the photovoltaic industry owing to the low‐cost, high substrate compatibility, rapid film‐formation, easy patterned, and scalable production. Some materials can be used to prepare high viscosity and long‐term stability of screen‐printing inks, including TiO_2_, NiO_X_, ZrO_2_, Al_2_O_3_, Carbon, Ag, and perovskite. Screen‐printing technique coats the substrate simply by rapidly sweeping the scraper on a patterned metal or polyester screen loaded with screen‐printing paste.^[^ ^35^ ^]^ The pattern area of the screen facilitates the transfer of the paste, while the surplus area blocks the paste transfer, resulting in the patterned printing of the film. This printing method can transfer paste onto various substrates, including both flexible and rigid ones, such as polyvinyl chloride (PVC), polyethylene terephthalate (PET), polyethylene naphthalate (PEN), polycarbonate (PC), ceramics, conductive glass, and aluminum sheets.^[^ ^36^, ^37^, ^38^, ^39^ ^]^ Therefore, screen‐printing technique is considerably suitable for the fabrication of various thin‐film‐based devices.

In photovoltaic applications, screen‐printing is primarily employed in printing patterned Ag electrodes for crystalline‐silicon photovoltaic cells (c‐Si PVs), and then in printing mesoporous TiO_2_ layer for dye‐sensitized solar cells (DSSCs).^[^ ^40^, ^41^, ^42^, ^43^, ^44^ ^]^ In the last decade, considering the advantages of screen‐printing technology in producing multi‐functional thin films, the concept of all screen‐printed PSCs has been proposed, which could greatly reduce the cost and time consumption of fabricating PSCs on a large scale. Actually, some functional layers in PSCs, such as mesoporous/compact electron transport layer and carbon electrode, have already been shown to be screen‐printable, but the screen‐printing of perovskite film is challenging due to the extremely low viscosity of perovskite precursor solution based on traditional organic solvents.^[^ ^45^, ^46^, ^47^, ^48^, ^49^ ^]^ This bottleneck has not been broken for a long time until recently, our group has developed an ionic liquid with tunable viscosity to use as a single solvent for perovskite precursors and realized the first all screen‐printed PSCs with decent PCE over 20%, demonstrating the great potential of screen‐printing technology in the field of PSCs.^[^ ^9^ ^]^ Therefore, considering the inspiring progresses being made, a comprehensive and systematic review of the application of screen‐printing technology in PSCs is highly desired in the community to further guide the low‐cost, large‐scale, and commercialization development of perovskite photovoltaics.

Herein, we dedicate to providing an overview of the advanced development of PSCs based on screen‐printing technology. We begin with the introduction of the technical principles and procedures of screen‐printing, based on which the superiorities of screen‐printing over other printing/coating methods are highlighted. After that, screen‐printing of the typical functional layers in PSCs are summarized, including hole blocking layer (HBL), electron transport layer (ETL), insulating layer, hole transport layer (HTL), perovskite layer, and counter electrode (CE). Subsequently, we summarize the state‐of‐the‐art strategies for improving the quality of screen‐printed functional layers to enhance their PSCs performance, including composition engineering, additive engineering, solvent engineering, and interface engineering. Finally, we give a critical outlook on the future development trend of large‐scale PSCs based on printing technology.

## 2 Screen‐Printing Technology

Printing technology has been in existence for over a century, tracing its roots back to ancient movable type printing method developed in China, which is also known as porous printing.^[^ ^50^ ^]^ Screen‐printing is one of the typical porous printing methods, and it is a versatile method that is capable of printing both continuous and discontinuous details with flexibility. During World War II, it was utilized to mass‐produce electronic circuitry as it could deposit patterned thick conductive materials that conveyed power without significant voltage loss, making it an ideal alternative to manually routing wires.^[^ ^51^ ^]^ In the “NASA era” of 1960s, the push for miniaturization of electronic devices prompted the use of screen‐printing technology for miniature electronic circuit printing. Since then, this technology has gained great popularity and become an integral part of electronic production with the introduction of resistors and dielectrics, leading to the onset of the ‘thick film’ period.

Screen‐printing method, with the characteristics of low‐cost, rapid prototyping, convenient fabrication, and versatility, can print specially‐made ink into complex patterns by utilizing a designed mesh, and this technique could transfer ink onto almost all kinds of substrates.^[^ ^29^ ^]^ The operation mechanism of screen‐printing machine is a round‐trip process, including filling the open area of mesh with the drag of the scraper during forward kinematics and transferring ink onto the surface of the substrate under a certain squeegee force during inverse kinematics. Through multiple round‐trip printing steps, stereoscopic composite structures perpendicular to the substrate surface can be readily manufactured. In recent years, screen‐printing has been widely applied in various miniaturized integrated circuits to reduce their large‐scale industrial costs,^[^ ^52^ ^]^ which greatly promotes the practical application of this classic ancient printing technique.

**Figure 1:**
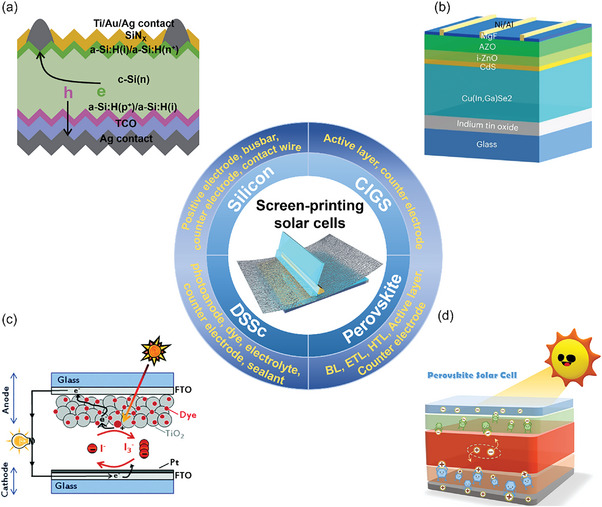
Application of screen‐printing technology in the field of solar cells. a) c‐Si PV. Reproduced with permission.^[^ ^53^ ^]^ Copyright 2021, Elsevier. b) Copper Indium Gallium Selenide (CIGS) solar cells. Reproduced with permission.^[^ ^54^ ^]^ Copyright 2023, Springer Nature. c) DSSc. Reproduced with permission.^[^ ^55^ ^]^ Copyright 2015, Royal Society of Chemistry. d) PSCs. Reproduced with permission.^[^ ^56^ ^]^ Copyright 2022, American Chemical Society.

In the field of photovoltaic application, screen‐printing method has been widely used in different generation devices from crystalline c‐Si PVs to CIGS solar cells, DSSCs, and PSCs (**Figure** **1**). In c‐Si PVs, the positive electrode, busbar, counter electrode, and contact wire are all printed by screen‐printing technology (Figure 1a), while in CIGS solar cells, the active layer and counter electrode are prepared by screen‐printing technology (Figure 1b). In DSSCs, screen‐printing technology can be used to prepare all the functional layers, including photonodes, dyes, electrolytes, counter electrodes, and sealants (Figure 1c). Inspired by the developed of all screen‐printing DSSCs, screen‐printing technology has been applied to the preparation of all functional layers of PSCs, including HBL, ETL, HTL, active layer, and CE. It has proven to be a reliable method for preparing modules (Figure 1d).

In this section, the theoretical models and key parameters of screen‐printing technology will be introduced, and the features of screen‐printing compared to other printing methods are also discussed.

### 2.1 Theoretical Models of Screen‐Printing

At the early stage, screen‐printing method has long been used in garment manufacture without knowing the scientific principle that governs it. Although some assumptions have been made at that time, they were proved to be incorrect and lack of prediction accuracy, leading to the stereotype that screen‐printing technique was an art rather than a science. To make good use of this technology in a wider field, establishing a theoretical model to accurately describe the whole screen‐printing process is highly desired. For screen‐printing method, the ink transfer during round‐trip screen‐printing process is considered to be the key governing factor that determines the quality of deposited layer, and thus researchers are making their efforts in developing a theoretical model to simulate the ink transfer process.

Riemer et al. first described the ink transfer process using an analytical model based on the Navier‐Stokes equation in cylindrical polar coordinates simplified for creeping flows.^[^ ^57^ ^]^ However, this model assumes that ink is injected exclusively by hydrostatic pressure, which ignores the adhesive and cohesive forces of the ink, and thus it fails to predict the total depositions of the ink. As a result, many researchers soon altered their attentions to investigate the in‐depth dynamics issues of the squeegee blade instead of ink, for example, the role of the squeegee blade during the squeegee blade's movement and the action of the squeegee blade in filling the screen based on computational fluid dynamics (CFD) simulations.^[^ ^58^ ^]^ These studies focused on the hydrodynamic conditions at the squeegee tip and were concerned with the role of associated pressure differentials in the screen‐to‐substrate transfer process, where the screen is considered as a permeable membrane (or a network of capillaries) and Darcy's law is used to solve the issue analytically.^[^ ^59^, ^60^, ^61^ ^]^

**Figure 2:**
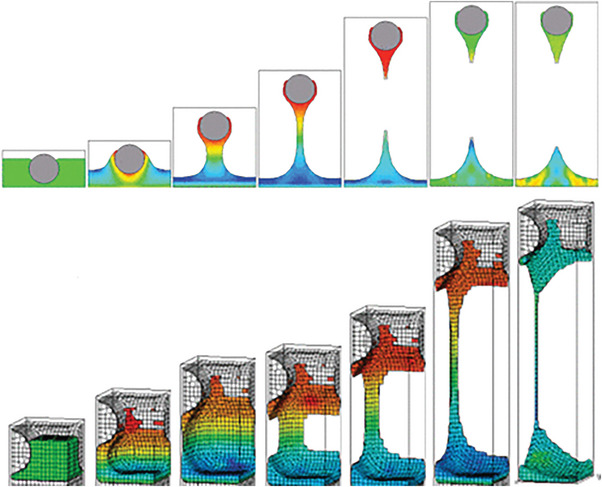
The 2‐D and 3‐D models behavior of ink during screen‐printing process. Reproduced with permission.^[^ ^63^ ^]^ Copyright 2013, Institution of Electrical and Electronics Engineers.

However, none of the above‐mentioned studies have addressed the essential underlying assumption that the squeegee action in filling the screen is actually related to the following emptying procedure. During the process, the majority of the ink paste would tend to stay on the screen, and because the squeegee has no effect once ink transferring begins, squeegee pressure and speed have nearly no effect on the process of ink transferring. Furthermore, gravity and the presence of air above the screen are also found to have no effect on the whole process. Instead, factors that influence the interaction forces between ink and substrate, such as viscosity, cohesive, adhesive forces, and pseudoplasticity of the ink and mesh angle relative to the substrate are the primary factors governing the total printing process. Messerschmitt et al. asserted that the cohesive forces of the ink, which is regulated by the surface tension of the liquid, are the primary driving force for screen‐printing, and a liquid bridge‐like structure forms when the screen and substrate are separated.^[^ ^62^ ^]^ The authors made important observations about the process of screen‐to‐substrate transferring, and it was proposed that adhesion, extension, flow, and separation are the four steps of ink transfer to the substrate. Thereafter, Abbott et al. expanded the process into a computer model.^[^ ^63^ ^]^ As shown in **Figure** **2**, the 2D and 3D geometric shapes of printing ink in screen‐printing are simulated, which is conducive to better understanding the separation process between printing ink and substrate to accurately predict the thickness and morphology of the printed film.^[^ ^63^ ^]^ According to this computational model, the volume of the ink liquid between the mesh and the substrate can be divided into 100 rectangular sections in two dimensions, and the capillary number and meniscus behavior can be calculated based on the ink's rheology. This model is shown to be well‐matched with experimental data for ink compression and inertia effects, and on the basis of the model, the follow‐up studies further focus on the process control of screen‐printing technique by optimizing the technique parameters.

### 2.2 Key Parameters of Screen‐Printing

**Figure 3:**
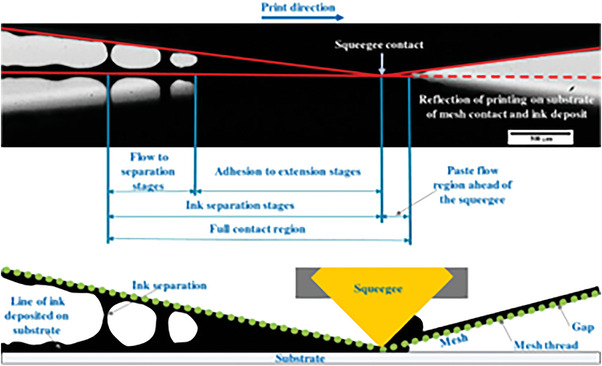
The cross‐sectional schematic diagram of the screen‐printing process. Reproduced with permission.^[^ ^64^ ^]^ Copyright 2020, Springer Nature.

As shown in **Figure** **3**, the typical screen‐printing process transfers ink onto a substrate surface via a mesh, during which the ink is applied on the mesh using a squeegee that is pushed across the screen. The ink is blocked at the regions of the mesh made impervious to ink using a blocking stencil, and it only fills up the open spots of the mesh).^[^ ^56^ ^]^ By repeating the process, multiple layers of ink can be successively applied on the substrate, allowing for precise control of layer thickness.

There are many steps involved in this screen‐printing process, and a number of factors will affect the quality of printed layers. These factors can be divided into four parameter groups: printer parameters (printing speed, printing pressure, squeegee shape, squeegee angle), stencil parameters (screen mesh, aperture size, screen thickness, screen processing technology), environmental parameters (temperature, humidity, air velocity), and ink parameters (viscosity, solid content, thixotropy, drying time, rheology).

Printer parameters determine the whole contact‐transfer process between the screen and the substrate, including the paste flow, adhesion, and separation stage, affecting the uniformity and smoothness of the wet film. Stencil parameters mainly affect the thickness of wet film and the quality and stability of annealed film. Environmental parameters can determine the quality of deposited films because environmental conditions play a decisive role in the rate and direction of solvent volatilization, which significantly affects the defects, flatness, and crystallinity of annealed films. Screen‐printing ink parameters determine the viscosity, fluidity, and dispersion of the ink, which have a great impact on uniformity and drying speed of the ink as well as the thickness of the deposited film. In sum, all these parameters have an influence on the film‐formation and patterning characteristics of screen‐printed film, and thus smart optimization of these parameters are the key to obtaining high‐quality perovskite film.

### 2.3 Superiorities of Screen‐Printing Over Other Printing Methods

High‐throughput large‐scale PSCs production requires a film deposition method with high volume and high speed,^[^ ^65^ ^]^ which is a fundamental technique basis toward industrialization upgrading of PSCs.^[^ ^66^ ^]^ Currently, blade coating,^[^ ^67^ ^]^ slot‐die coating,^[^ ^68^ ^]^ D‐bar coating,^[^ ^69^ ^]^ inkjet printing,^[^ ^70^ ^]^ spray printing,^[^ ^71^ ^]^ and screen‐printing,^[^ ^72^ ^]^ are some of the most extensively studied scalable methods for printing functional layers on rigid/flexible substrates. These methods show different printing features according to the contact method between ink and substrate, and screen‐printing method possesses its own superiorities.

**Figure 4:**
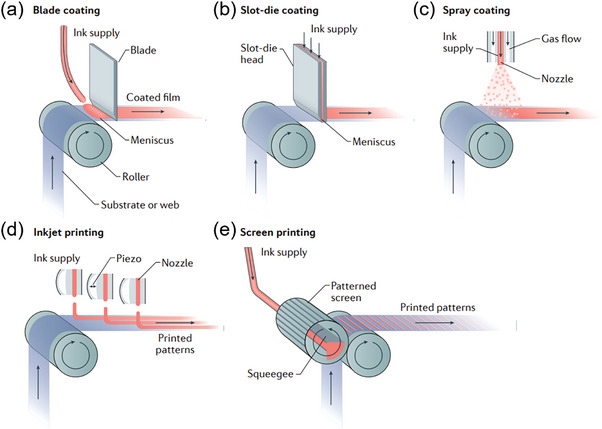
Schematics of production process of some typical printing techniques. a) Blade coating. b) Slot‐die coating. c) Spray coating. d) Inkjet printing. e) Screen‐printing. Reproduced with permission.^[^ ^29^ ^]^ Copyright 2018, Springer Nature.

Blade coating, slot‐die coating, and D‐bar coating are typical meniscus coating, and they deposit thin films in a manner of line–surface stretching. The “meniscus” means to form a smooth perovskite surface to rate the evaporation of the solvent, which is influenced by the geometry of the meniscus, substrate temperature, gaping, and speed. As shown in **Figure** **4**a, blading coating method utilizes a coating instrument to spread the perovskite ink across the surface of a substrate.^[^ ^66^ ^]^ While slot‐die coating is similar to blade coating but with a different coater, which has two dies to distribute the perovskite solution of interlayer over a substrate (Figure 4b). During the coating process, the coating solution fills the gap between the die and the substrate and then forms the coating bead by the meniscus. The deposition process based on slot‐die coating is greatly affected by solvent properties (viscosity) and mechanical parameters (coating speed and gap). For D‐bar coating, it uses a cylindrical wire bar to load ink on the substrate. The ink within the wire gap persists on the substrate during the coating process, forming wet films, and thus the thickness of a wet film can be precise control by the gap depth with high reproducibility.

While spray coating, inkjet printing, and screen‐printing deposit thin films in a manner of point–line connecting. As shown in Figure 4c, spray coating can produce a large amount of tiny liquid droplets from ink through an air nozzle to a heated substrate. Owing to the continuous dynamics of the spray process, compact and smooth layer can be fabricated. At present, pneumatic spraying (droplets are bigger but uneven) and ultrasonic spraying (droplets are smaller but uniform) are widely used in deposition of large‐scale perovskite films.^[^ ^73^ ^]^ For inkjet printing, the fluid is maintained at ambient pressure, and a piezo transducer is used to create a drop only when needed (Figure 4d). Such systems were first described by Hansell in the late 1940s. For successful drop formation, the transfer of kinetic energy from the transducer to the ink must be large enough to overcome the surface tension at the nozzle. An excitation pulse in the range of ≈1 to 100 µs creates a volumetric change in the fluid, which in turn induces different pressure waves and undergoes constructive and destructive interference traveling through the print‐head.

For screen‐printing method, a hydrodynamic pressure is created in the wedge of print paste that exists between the squeegee and the mesh in the printing procedure (Figure 4e). Because the included angle θ between the direction of sequential droplets and the direction of apparatus movement ranges from 0° to 180°, the quality of printed pattern directly depends on the distribution of each ink droplet with the manner of point–line–surface. It can be seen that the film‐forming mode of the screen‐printing printing technique compared to other techniques makes it much easier to realize a patterned printing process with a high degree of freedom,^[^ ^23^ ^]^ which is beneficial for fabricating pattern‐designed PSCs on substrate with various sizes and shapes that can meeting the needs of different application scenarios.

## 3 Screen‐Printing Functional Layers in PSCs

Owing to its low cost and scalability, the potential of screen‐printing technology in the fabrication of PSCs is deeply investigated, especially in the large‐area PSC modules. To date, all functional layers of PSCs could be fabricated by screen‐printing method, including hole blocking layer, electron transport layer, insulating layer, hole transport layer, perovskite layer, and electrode layer (Carbon or Silver). Notably, the screen‐printing of most functional layers in PSCs has been well‐studied, but the screen‐printing of perovskite layer is just realized recently by our group via the development of ionic liquid as a solvent to produce perovskite ink.^[^ ^23^ ^]^ Currently, the development of printing ink and optimization of printing parameters are still the main research subjects in optimizing the performance of screen‐printed PSCs, including solvent components, chemical composition, and printing parameters. In this section, the advanced development and control strategies of screen‐printing functional layers in PSCs will be discussed.

### 3.1 Hole Blocking Layer

In planar PSCs with the sandwich structure, the HBL is necessary to prevent the hole carrier transfer but promote the electron carrier extraction to electrode, avoiding harmful recombination of the carriers and improving the photoelectric performance of the device. As a result, the HBL is normally a compact thin film with ≈30–40 nm thickness and high electron conductivity, Such HBL can be prepared by many methods, including spin‐coating, atomic layer deposition (ALD), electrodeposition, spray coating, and screen‐printing.^[^ ^74^ ^]^

**Figure 5:**
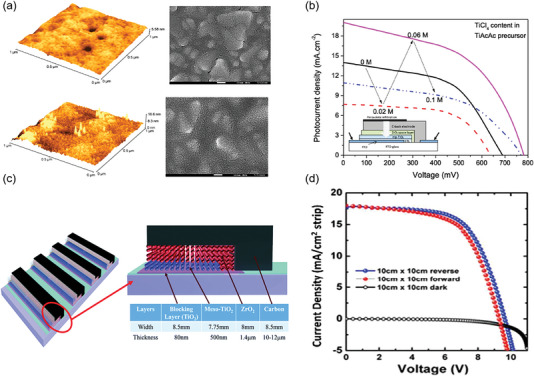
a) AFM topography and top‐view SEM of c‐TiO_2_ hole‐blocking layers prepared with the content of 0 m and 0.06 m TiCl_4_. b) *J–V* curves of solar cells with blocking layers prepared from TiAcAc precursors with and without TiCl_4_. Reproduced with permission.^[^ ^74^ ^]^ Copyright 2021, Elsevier. c) Schematic diagram of the screen‐printed PSC module and the thickness of each functional layer. d) *J–V* curves of PSC modules with an active area of 70 cm^2^ (10 cm × 10 cm). Reproduced with permission.^[^ ^77^ ^]^ Copyright 2016, Royal Society of Chemistry.

For the screen‐printing method, several HBLs, such as TiO_2_ and ZnO,^[^ ^75^, ^76^ ^]^ can be stably prepared with low roughness and uniform thin films. The screen‐printed compact TiO_2_ (c‐TiO_2_) is currently the most commonly‐used technique in large‐scale preparation of PSCs due to low‐cost, facile, and stable of the process. However, early work found that screen‐printed c‐TiO_2_ layer based on pristine TiO_2_ ink show holes and cracks morphology, As shown in **Figure** **5**a, adding an appropriate amount of TiCl_4_ into HBL printing ink reduces the cracking of screen‐printing films, which is attributed to the special texture of very fine agglomerates (≈30 nm) that hinders the merging of small agglomerates into larger agglomerates and cracks. Figure 5b shows the *J–V* curves of screen‐printed devices with BLs prepared form different content of TiCl_4_ in TiAcAc, and the best results were obtained at 0.06 m TiCl_4_, which is related to the conductivity of blocking layers.^[^ ^72^ ^]^ As shown in Figure 5c, Anish Priyadarshi et al. prepared a large‐area (10 cm × 10 cm) fully screen‐printed PSC module with low c‐TiO_2_ resistance by TiCl_4_ pretreatment before and after screen‐printing of c‐TiO_2_.^[^ ^77^ ^]^ The printing mesh was designed to produce the module with maximum active area (dead line width is 3 mm, active line width is 7.75 mm) while preventing short circuits among adjacent strips. After optimization, the PCE of PSC modules achieved 10.46% and 10.74% with 31 and 70 cm^2^, respectively (Figure 5d).^[^ ^75^ ^]^ Moreover, the PCE decay of the module was <5% after ambient aging for >2000 h. At the same time, the authors highlighted that the high‐efficiency and stable PSC modules depended on the quality of the dense layer and the infiltration of the perovskite ink, and the schematic diagram of layer‐by‐layer fabrication of screen‐printed perovskite devices. In 2017 years, Anish Priyadarshi et al. significantly improved the performance of the fully screen‐printed PSCs by the double‐printing of the HBL‐TiO_2_ while increasing the thickness of the ZrO_2_ layer, and this type of device was designed to facilitate the manufacturing process by replacing the mesoporous TiO_2_ (m‐TiO_2_) layer with a thick insulating layer. The repeated screen‐printed BL‐TiO_2_ showed a significant advantage in passivating defects on the surface of FTO.^[^ ^78^ ^]^ In addition, it was found that the concentration of TiCl_4_ for the post‐treatment affected the surface morphology and carrier transport properties of c‐TiO_2_, and an excessively high concentration of TiCl_4_ lead to forming the broken layer on the surface of the c‐TiO_2_, which increases harmful surface defects. After systematic optimization of processes, the screen‐printed PSCs gave an optimized efficiency of 9.69%.

### 3.2 Electron‐Transport Layer

There are many mature ETL deposition techniques, which can be divided into two categories, which are physical deposition methods and chemical deposition methods. Physical deposition methods include spin coating, blade coating, screen‐printing, electro hydrodynamic deposition, thermal spray, physical vapor deposition, and pulsed laser deposition, while chemical deposition methods include chemical vapor deposition, atomic layer deposition, thermal oxidation, chemical bath deposition, spray pyrolysis, electrochemical deposition.^[^ ^79^, ^80^ ^]^ Among them, screen‐printing and spray coating are the typical representative of printing techniques and coating techniques suitable for ETL deposition with large‐scale production in contrast to coating processes, where the paste across through the substrate surface by spray nozzle, screen‐printing applies squeezing pressure to transfer the paste from the pattern screen to the substrate surface.^[^ ^81^, ^82^ ^]^ Therefore, screen‐printing has significant advantages in reducing raw material waste and rapidly preparing thin films. What's more, the porosity and transparency of the sintered ETL film can be more accurately controlled by screen‐printing method through i) printing parameters such as wire diameter, mesh count, and printing pressure and ii) the proportion/solid content of additives and Ti/Sn source to control the slurry viscosity. As a result, these distinct properties of screen‐printing method have great amplification advantages to provide high‐quality large‐area ETL films.^[^ ^83^ ^]^

In 2009 years, screen‐printed m‐TiO_2_ as an n‐type electron‐acceptor was first applied in perovskite‐based DSSCs.^[^ ^84^ ^]^ Kojima et al. fabricated a layer of CH_3_NH_3_PbX_3_ (X = Br, I) perovskite as dye molecules on the surface of m‐TiO_2_.^[^ ^85^ ^]^ By adjusting the thickness of the screen‐printed m‐TiO_2_ film, the highest PCE of 3.81% was obtained in CH_3_NH_3_PbI_3_ perovskite. This efficiency is significantly higher than those DSSCs based on inorganic sensitizers and quantum dots at the time. However, due to the incomplete filling of m‐TiO_2_ by the perovskite precursor solution, the performance of mesoscopic perovskite devices suffered an undesired decline. To solve this, Rossi et al. increased the printing speed from 110 to 220 mm s^−1^ and the printing gap from 2.2 to 3.2 mm during screen‐printing of m‐TiO_2_. The increased printing speed and printing angle are conducive to obtaining uniform m‐TiO_2_ films since the coffee ring effect was suppressed and average roughness (Ra)/average roughness (peak roughness Rp) were reduced.^[^ ^86^ ^]^ Finally, the A4‐sized Carbon‐PSCs (C‐PSCs) module only consume 1.6 mL of perovskite solution, and the screen‐printed device gained a PCE of over 6%. The improved device efficiency is attributed to better interfacial contact and filling of perovskite in m‐TiO_2_.

In 2014, Mei and co‐workers reported a continuous screen‐printing hole‐transporting layer‐free PSCs with the device structure of FTO/c‐TiO_2_/m‐TiO_2_/(5‐AVA)_x_(MA)_1‐x_PbI_3_/m‐ZrO_2_/Carbon.^[^ ^87^ ^]^ This novel fully printing device achieved a PCE of over 10%, avoiding the use of expensive metal electrodes (gold, silver) and hole transport layer (Spiro‐OMeTAD) by adopting graphite as CE. Note that perovskite layer was prepared by spontaneous infiltration of the perovskite precursor solution into m‐TiO_2_ passing through the graphite CE layer. High‐resolution TEM images showed that 5‐ammonium valeric acid (5‐AVA)‐based (5‐AVA)_x_(MA)_1‐x_PbI_3_ perovskite can better cover the surface of TiO_2_ in a uniform manner compared to MAPbI_3_. The improved perovskite crystal quality and the increased perovskite loading in m‐TiO_2_ may be due to the template effect of 5‐AVA that affects the crystallization and growth of perovskite crystal.

**Figure 6:**
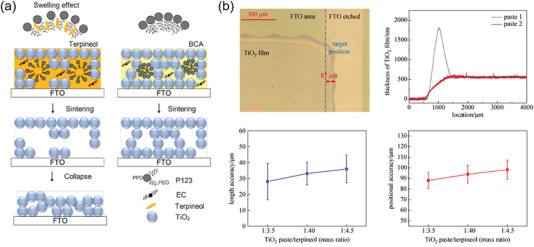
a) Illustration of the formation mechanism of highly porous TiO_2_ films by using copolymer P123 as a pore‐adjusting agent. Reproduced with permission.^[^ ^88^ ^]^ Copyright 2016, American Chemical Society. b) Characterizations of positional accuracy, thickness variation, length accuracy, and positional accuracy of the screen‐printing m‐TiO_2_ films. Reproduced with permission.^[^ ^89^ ^]^ Copyright 2019, Springer Nature.

The above pioneer work indicates that the pore‐filling of perovskite inside m‐TiO_2_ will significantly affect the charge extraction at the TiO_2_/CH_3_NH_3_PbI_3_ interface and the performance of solar cells. However, the use of graphite as CE hinders the sufficient permeation of perovskite precursor solution. To solve this, Han's group explored the effect of two counter‐electrodes prepared by graphite with different morphologies on perovskite filling in m‐TiO_2_, including spheroidal graphite CE (SG‐TiO_2_) and flake graphite CE (FG‐TiO_2_).^[^ ^84^ ^]^ Compared with the bare TiO_2_ film, the CH_3_NH_3_PbI_3_ filling inside the SG‐TiO_2_ was more uniform than that of the FG‐TiO_2_, which was attributed to the loose structure of the spheroidal graphite with spherical morphology. Furthermore, smooth morphology of SG was more conducive to filling the perovskite solution from the carbon electrode into the m‐TiO_2_. As a result, this SG‐TiO_2_ device showed high stability and PCE of 6.64% with higher FF. In 2016, Shao et al. used a novel slurry based on a polymer additive to prepare m‐TiO_2_ thin films with highly porous and large pore‐size structures.^[^ ^88^ ^]^ Specifically, by adjusting the amount of copolymer P123 modifier, a screen‐printing paste with 2‐butoxyethyl acetate as solvent was prepared, which was used to prepare m‐TiO_2_ films with a pore size of 16.0−34.2 nm and a porosity of 57.1−73.5% (**Figure** **6**a). The screen‐printed m‐TiO_2_ film with high porosity is found to avoid the accumulation of carriers and increased the electron injection rate at the interface, and a hysteresis‐free PSC device with an efficiency of 15.47% can be obtained.

Although screen‐printing technology has been used to deposit micron‐sized m‐TiO_2_ films before, it is still challenging to screen‐printing nano‐sized m‐TiO_2_ films with precisely controlled and uniformly distributed hole sizes. As shown in Figure 6b, Wan et al. controlled the morphology and thickness of m‐TiO_2_ by adjusting the solute content of the printing paste and printing parameters and comprehensively expounded the relationship between the thickness of m‐TiO_2_ film and printing parameters, including solid content, viscosity, printing speed, printing pressure, and ambient temperature. The edge effect of m‐TiO_2_ films was proposed to be related to the aggregation effect caused by the long‐term storage of the slurry, and the elimination of the adverse effect was beneficial to the large‐scale application of screen‐printed PSCs.^[^ ^89^ ^]^ Unfortunately, PSCs fabrication was not reported in the work.

**Figure 7:**
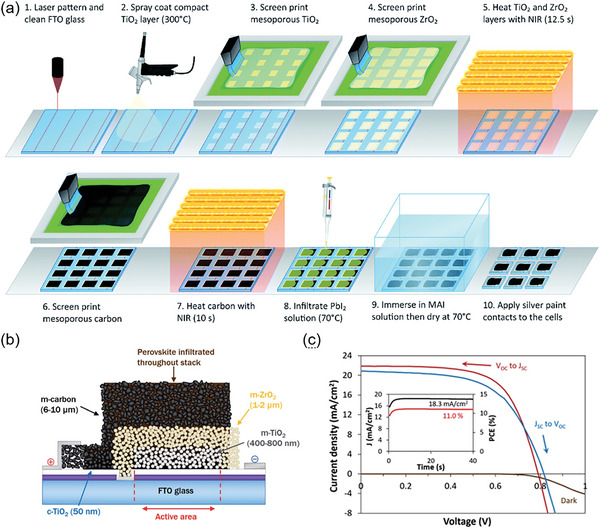
a) Schematic of the screen‐printing processing line of PSC based on the Near‐infrared (NIR) technology. b) Schematic of a screen‐printed PSC device based on NIR technology. c) *J–V* curses of champion PSCs by NIR process, inset: stabilized current and PCE measurement. Reproduced with permission.^[^ ^45^ ^]^ Copyright 2017, Royal Society of Chemistry.

Although the well‐controlled screen‐printed ETL can produce efficient PSCs, the fabrication of m‐TiO_2_ layer requires continuous high‐temperature sintering to achieve complete phase‐transformation of TiO_2_ crystals, which is a very expensive process and is not conducive to continuous device production. In the sintering process, the TiO_2_ first forms a metastable brookite phase, which can be basically transformed into a relatively stable anatase at ≈300 °C. In addition, with the increasing temperature, the polymer adhesive in the TiO_2_ ink will be completely decomposed, thus forming a TiO_2_ layer with a mesoporous structure. To reduce the processing temperature, Baker et al. used the near‐infrared (NIR) heating process to treat the screen‐printing m‐TiO_2_ film. As shown in **Figure** **7**a, the overall high‐temperature process was only 22.5 s with a linear speed of 2 m min^−1^, which reduced the total time of preparing a complete CE‐based PSCs to <1 h.^[^ ^45^ ^]^ Besides, the residual stress caused by rapid heating and cooling treatment was solved through device structure optimization and heating uniformity improvement (Figure 7b). This technology replaced the traditional time‐consuming high‐temperature sintering process and obtained CE‐based PSCs with PCE> 11% (Figure 7c). DI.G.F et al. developed a flexible PSCs module based on screen‐printing technology, where a low‐temperature UV‐irradiation technique was successfully applied to fabricate m‐TiO_2_ layer.^[^ ^90^ ^]^ In this process, the UV‐irradiation treatment decomposed the organic binder (cellulose) in the m‐TiO_2_ film and significantly improved the interparticle bonding and phase transition between TiO_2_ grains. A flexible perovskite module with an efficiency > 4% was prepared by optimizing the formula of m‐TiO_2_ paste, the screen‐printing parameters, and the laser parameters.

### 3.3 Insulating Layer

The insulating layer, also named as the spacer layer, is used to prevent direct contact between the ETL and the carbon‐based CE in PSCs, and it also allows the carrier separation, prevents the holes migration to the anode, and suppresses the excited electron‐hole pair recombination. A high‐quality insulating layer is instrumental in improving the performance of a hole‐free device. Screen‐printing ZrO_2_ is the most used insulating layer for interface engineering in screen‐printed PSCs. First “HTL‐free” device reported by Han et al. used only inorganic materials as the device support layer, and the ZrO_2_ layer is screen‐printed on the bottom of the carbon electrode as an insulating layer, which can effectively prevent the recombination of carriers at the carbon electrode.^[^ ^87^ ^]^ The cationic perovskite (5‐AVA)_x_MA_1‐x_PbI_3_ could effectively boost the PCE of the screen‐printed HTL‐free PSCs to 12.8%. Liu et al. fabricated a m‐TiO_2_/m‐ZrO_2_/carbon device and controlled the thickness of the insulating layer, which mainly depended on the printing parameters.^[^ ^91^ ^]^ PSCs without ZrO_2_ films exhibited rather poor optoelectronic properties, while when extremely thin ZrO_2_ was introduced, the device performance was significantly improved, where the highest PCE of >10% were obtained based on the devices with micron‐scale thickness of ZrO_2_ film. However, when the thickness of ZrO_2_ film continued to increase, the screen‐printed device showed decreased *V*oc and FF, which is because the thick film reached the limit of carrier diffusion length.

**Figure 8:**
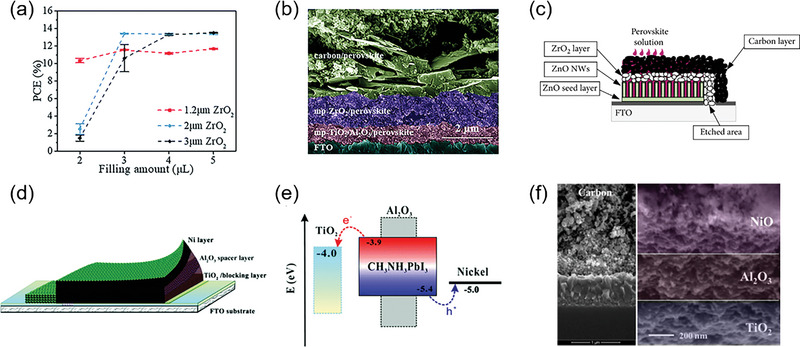
a) The average performance of screen‐printed PSCs with different thicknesses of ZrO_2_ films and b) cross‐sectional SEM image of the device. Reproduced with permission.^[^ ^92^ ^]^ Copyright 2018, Royal Society of Chemistry. c) Schematic of screen‐printed PSCs with the structure of ZnO seed layer/ZnO NWs/ZrO_2_. Reproduced with permission.^[^ ^94^ ^]^ Copyright 2019, Hindawi. d) Illustration and e) the corresponding energy levels of the screen‐printed PSCs with the structure of FTO/c‐TiO_2_/m‐TiO_2_/Al_2_O_3_/Ni. Reproduced with permission.^[^ ^95^ ^]^ Copyright 2015, Royal Society of Chemistry. (f) Cross‐sectional SEM image of the screen‐printed PSCs with the structure of TiO_2_/Al_2_O_3_/NiO/C (filled with MAPbI_3_). Reproduced with permission.^[^ ^96^ ^]^ Copyright 2015, Elsevier.

Usually, a 2–3 µm m‐ZrO_2_ layer is required, but this leads to increased perovskite precursor loading capacity and poor reproducibility. Wang et al. used a very thin layer of Al_2_O_3_ (spray pyrolysis) to significantly reduce the thickness of m‐ZrO_2_ layer down to 1.2 µm) without any loss of device performance. As shown in **Figure** **8**a,b, The introduction of the Al_2_O_3_ insulating layer retards the charge recombination between TiO_2_ and carbon electrodes by down‐shifting the conduction band minimum (CBM), which could reduce the thickness of m‐ZrO_2_ layer and improve the carrier transport properties.^[^ ^92^ ^]^ Priyadarshi et. al found that PSCs based on bilayer c‐TiO_2_/ZrO_2_ showed sufficiently comparable device performance to standard those based on m‐TiO_2_ layer, and a thicker ZrO_2_ layer (2100 nm) can further replace the bilayer c‐TiO_2_ (500 nm)/ZrO_2_ (1000 nm). As a result, the fully screen‐printed device could reduce cycle preparation time and thermal energy.^[^ ^93^ ^]^ In addition, double‐layer ZrO_2_ (nanoparticles and microparticles) have been developed (Figure 8c), and the double layer shows both insulation and light scattering effects. Moreover, this double‐layer ZrO_2_ thin film was found to improve the growth and light absorption of perovskite crystals, and its high surface roughness improves interfacial contact and device performance.^[^ ^94^ ^]^

In addition to ZrO_2_, Al_2_O_3_ was also widely deposited on the surface of m‐TiO_2_ ETL serving as an insulating Layer. Ku et.al developed a PSC device with a unique mesoporous nickel layer on Al_2_O_3_ insulating layer to make the CH_3_NH_3_PbI_3_ perovskite precursor penetrate deeper into the m‐Al_2_O_3_ layer (Figure 8d). They used a ≈2 µm‐thick mesoporous nickel layer as the CE of the device, and the insulating layer Al_2_O_3_ could separate the nickel and TiO_2_ layer to ensure that there is no risk of direct contact between the two electrodes.^[^ ^95^ ^]^ The screen‐printed Al_2_O_3_ spacer and TiO_2_ layers were optimized to be 1 µm and 500 nm, respectively. Since the thickness of each film in the FTO/c‐TiO_2_/m‐TiO_2_/Al_2_O_3_/Ni mesoporous scaffold is very thin, CH_3_NH_3_PbI_3_ can be easily filled in the m‐TiO_2_ holes by a two‐step method, which is beneficial for obtaining high‐performance devices. Figure 8e shows the energy level structure diagram of the device. In the following section 3.4, we will introduce the Ni counter electrode of this article. Cao et al. proposed a mesoscopic scaffold of TiO_2_/Al_2_O_3_/NiO/carbon quadruple‐layer to fabricate fully screen‐printed PSCs and the porous Al_2_O_3_ layer was applied between TiO_2_ and Nickel oxide (NiO) (Figure 8f).^[^ ^96^ ^]^ In this device structure, both NiO and Al_2_O_3_ layers can be used as spacers to prevent carrier recombination at the interface of TiO_2_ and carbon CE. After optimizing the thickness of Al_2_O_3_ layer, this quadruple‐layer fully screen‐printed PSCs based on MAPbI_3_ achieved >15% PCE under 100 mW cm^−2^ AM 1.5G solar irradiation.

### 3.4 Hole‐Transport Layer

The most efficient PSCs currently use Spiro‐oMeTAD as the HTL, which can form an efficient carrier transport system with typical metal oxide inorganic ETLs.^[^ ^97^ ^]^ However, Spiro‐MeOTAD is extremely expensive and not stable, and replacing materialist with p‐type metal oxide materials are beneficial to greatly reduce device cost and improve device stability. Compared to Spiro‐OMeTAD, NiO is an inexpensive p‐type metal oxide with tunable optoelectronic properties, and it is abundant in nature and can be synthesized on a large scale by a simple method.^[^ ^98^ ^]^ When inorganic NiO was combined with inexpensive commercial carbon materials, the fabrication cost of printing devices could be greatly reduced due to the removal of expensive metal electrodes and organic HTL, which can increase the competitiveness of printing modules.^[^ ^99^ ^]^ Typically, solution‐processed c‐NiO is often used as the HTL layer in inverted PSCs by spin coating, which can significantly improve the charge extraction rate and carrier lifetime. Moreover, due to the better thermal and chemical stability of NiO, the lifetime of photovoltaic devices can be significantly improved. In 2015, Xu et al. reported that m‐NiO prepared by doctor blade coating method can effectively accelerate the hole extraction at the interface by lowering the hole Fermi level, leading to improved device performance.^[^ ^99^ ^]^

**Figure 9:**
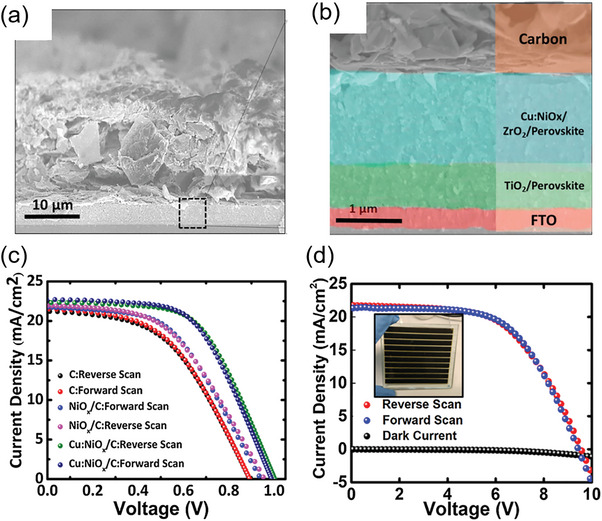
a) Low magnification and b) High magnification of cross‐sectional SEM images of the perovskite/carbon solar cell with Cu:NiO_X_ interlayer. c) Forward and reverse scan of device with Cu:NiOx layer with an aperture area of 0.8 cm^2^. d) Forward and reverse scan of device with Cu:NiOx layer with an aperture area of 70 cm^2^ SEM image of NiO_X_. Reproduced with permission.^[^ ^101^ ^]^ Copyright 2019, Elsevier.

Although optimization routes for NiO materials have been developed during the past few years, the screen‐printing of NiO layer with high carrier transport characteristics is still challenging because serious recombination at the interface results in degraded device performance. To solve the problem, Liu et al. modified the NiO nanosheet paste by mixing ethyl cellulose solution in a mixture of thanol, terpineol, and NiO nanosheet powder, and well‐dispersed NiO nanosheet paste can be obtained after detaching ethanol by rotary evaporation.^[^ ^100^ ^]^ It is shown that in screen‐printed PSCs with p‐i‐n configuration, the screen‐printed NiO nanosheet layer in the TiO_2_/ZrO_2_ composite structure could significantly improve the carrier collection efficiency and prolong the carrier lifetime within the device, thereby improving the overall performance of the device. Later, Bashir et al. reported the doping of NiO_X_ by Cu to improve the hole conductivity of NiO_X_ nanocrystals (Cu:NiO_X_).^[^ ^101^ ^]^ More specifically, 5% Cu‐doped NiOx were synthesized from the raw materials of NiCl_2_·6H_2_O and CuCl_2_·6H_2_O by chemical precipitation method, and then the preliminary products were mixed with terpineol and ethylcellulose and processed into a paste for the use of screen‐printing technology. Compared to prismatic NiO_X_, Cu:NiO_X_ nanostructures have higher surface tension and surface energy, which will increase the aggregation trend and the infiltration capacity of the ink. As shown in **Figure** **9**a,b, they then fabricated a PSC module with the device structure of FTO/c‐TiO_2_/m‐TiO_2_/ ZrO_2_/Cu:NiO_X_/Carbon, in which all functional layers could be fabricated by a screen‐printing method except for the perovskite layer was prepared by blade coating the solution that infiltrated from the surface of carbon electrode. It is found that screen‐printed Cu:NiO_X_ HTL could enhance the migration rate of charge carriers from perovskite active layer to carbon CE, helping to ascent device performance and stability of large‐area PSCs. After optimization, it is shown that the introduction of an 80 nm Cu:NiO_X_ mesoporous layer greatly increased the photoelectric performance of PSCs due to the excellent interfacial contact condition and increased hole extraction efficiency. As shown in Figure 9c, the screen‐printed device with Cu:NiOx interlayer shows a higher PCE with a high *V_OC_* of 1.00 V, *J_SC_* of 22.15 mA cm^−2,^ and FF of 0.57 leading to the PCE of 12.79% (active area = 0.8 cm^2^) compared to devices with NiOx and without any additional interlayer. As shown in Figure 9d, they further fabricated a large‐area rigid module with an effective area of ≈70 cm^2^ on a 100 cm^2^ substrate, finally achieving a PCE of 12.1%.

Since mesoporous layers are usually prepared before perovskite films and dense layers after them, challenges that may arise from the preparation process of c‐NiO_X_ and slurry composition for perovskite films must be overcome. The colloidal dispersion of ligand‐off NiO nanoparticles (NPs) has been proven not to destroy the perovskite layer by choosing an appropriate solvent with a spin coating method or blade coating.^[^ ^99^ ^]^ However, the low‐viscosity ink still cannot be used for screen‐printing process. If a layer of c‐NiOx is to be printed on the surface of the perovskite layer, the nanoparticles are required to have excellent dispersion ability in a high‐viscosity environment, and the volatilization of the solvent during the thermal deposition of c‐NiOx cannot destroy the perovskite. According to our best known, there is no such printing ink has been developed.^[^ ^102^, ^103^ ^]^

### 3.5 Perovskite Layer

Perovskite thin films could be fabricated on a small area (<1 cm^2^) mesoscopic PSCs by various methods, such as spin coating, blade coating, slot‐die coating et, and they all can produce efficient PSCs with high PCE. However, when fabricating amplified PSCs, the perovskite films prepared by those methods show poor homogeneous crystallization on large‐area substrates, leading to PSCs with low efficiency and poor stability.^[^ ^104^, ^105^, ^106^, ^107^ ^]^ Fortunately, screen‐printing technology with excellent printing freedom and patterning ability perfectly meets the industrial requirements of large‐area PSCs, because each ink droplet can be evenly distributed on the substrate to improve the uniformity and homogeneous permeation of perovskite solution. However, to realize the screen‐printing of perovskite film, the main challenge is that the typical organic solvent cannot prepare perovskite precursor solution with high stability and tunable viscosity for homogeneous ink transfer in screen‐printing process.

**Figure 10:**
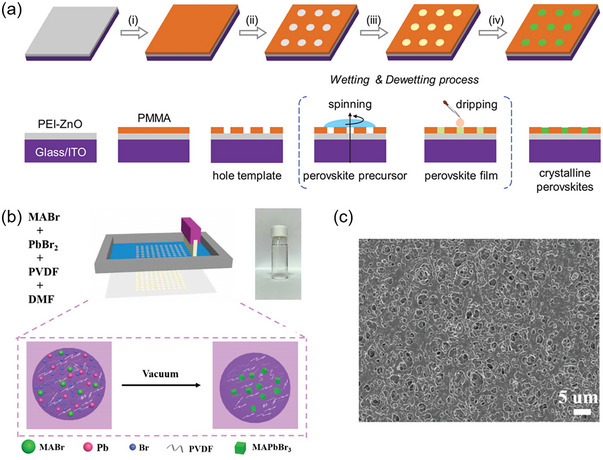
a) Schematic illustration of the patterning preparation of perovskite arrays. Reproduced with permission.^[^ ^109^ ^]^ Copyright 2020, Wiley‐VCH. b) Schematic illustration of screen‐printing MAPbBr_3_/PVDF layer. c) SEM image of screen‐printing perovskite film. Reproduced with permission.^[^ ^110^ ^]^ Copyright 2022, Institute of Physics.

At the early stage, researches focus on optimizing the ink transfer process when fabricating perovskite layer. Meroni. S. et al. developed a robotic mesh method (RbM) similar to screen‐printing technology, which can overcome the shortcoming of uneven infiltration of perovskite precursor solution based on organic solvents.^[^ ^108^ ^]^ An automatic dispenser and screen mesh were cooperated, which divided the perovskite solution into more drops to improve the homogeneous infiltration of perovskite solution. X‐ray diffraction and Raman spectroscopy showed that the distribution of perovskite nanocrystals in the mesoporous ETL was more uniform than that of the drop‐casting method. As a result, printed PSCs with >9% PCE can be fabricated. However, the ink distribution only relied on gravity, and thus RbM method was obviously not a mature scheme for wet‐films transferring. Wang et al. developed a wettability‐guided quasi‐screen‐printing method to prepare patterned perovskite film.^[^ ^109^ ^]^ As shown in **Figure** **10**a, the electron‐beam lithography (EBL) was used to exploit surface‐energy polymethyl methacrylate (PMMA) patterns to act as the “mesh” for screen‐printing process, and the perovskite arrays with controlled physical space were precisely fabricated by this quasi‐screen‐printing method based on a wetting/dewetting mechanism. Specifically, first, ZnO nanocrystals were spin‐coated on the surface of ITO as an electron transport layer, and a thin layer of PEI (polyethylene oxide) was coated on the surface of ZnO layer to adjust the wettability and work function to obtain a modified PEI‐ZnO composite film. Subsequently, a layer of PMMA film treated by standard EBL method was prepared on the surface of PEI‐ZnO as the initial screen‐printing “template”, and the MAPbI_3_ precursor solution was spin‐coated on the surface of patterned template to prepare patterned perovskite film. This work is a meaningful exploration of the preparation of patterned perovskite films, and the prepared film was successfully applied to the currently driven displays with large‐area perovskite array films.

To realize the screen‐printing of perovskite layer, the most effect strategy is to optimize the characteristics of perovskite ink. As shown in Figure 10b, Shan et al. added long‐chain polymer polyvinylidene difluoride (PVDF) as additives into the perovskite precursor solution to adjust the characteristics of ink and the morphology of the film.^[^ ^110^ ^]^ Specifically, the screen‐printing ink was prepared by mixing methylammonium bromide (MABr), lead bromide (PbBr_2_), and PVDF in organic solvent DMF (dimethylformamide), which can be stored stably for several months. The screen‐printed MAPbBr_3_/PVDF composite film need to be treated in a vacuum to remove excess DMF, and due to the local spatial effect, the perovskite nanocrystals were limited to grow in the polymer matrix, realizing the patterned preparation of porous perovskite films. As shown in Figure 10c, the morphology with intensive holes was obviously conducive to the preparation of perovskite thin films with high photoluminescence quantum yield (PLQY). Song et al. prepared the CsPbBr_3_/Cs_4_PbBr_6_ composite perovskite ink with high viscosity by ligand‐assisted precipitation at room temperature.^[^ ^111^ ^]^ By adjusting the composition and proportion of resin components, a perovskite ink with >2000 mPa·s viscosity and 100 s/100 µm drying rate was achieved, which enabled the screen‐printing of CsPbBr_3_/Cs_4_PbBr_6_ composite perovskite film. However, these methods could only produce screen‐printed perovskite nanocrystals because of the introduction of polymer or resin, which were not suitable for fabricating PSCs. Chu et al. proposed to use a two‐step process to prepare perovskite film, where PbO film was first deposited by screen‐printing method and then directly immersed in CH_3_NH_3_I solution to form CH_3_NH_3_PbI_3_ nanocrystalline thin film.^[^ ^112^ ^]^ They found that the concentration of CH_3_NH_3_I substantially affected the grain size of films, and concluded that this method could achieve low‐cost, high‐throughput printing process of perovskite films. However, the above strategies are only compromise solutions, and the key problem is not solved, that is, organic solvents‐based perovskite ink cannot be directly used for screen‐printing technique due to its low viscosity and stability.

**Figure 11:**
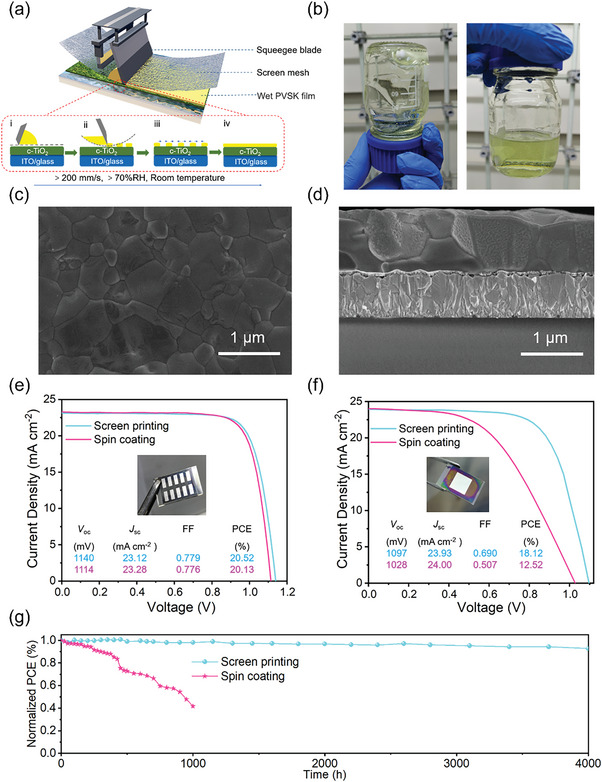
a) Diagram of the screen‐printing method for the deposition of perovskite thin films. b) Demonstration of high viscosity properties of ionic liquid MAAc. c) Surface and d) cross‐sectional SEM images of screen‐printing perovskite thin films based on MAAc. e) *J–V* curves for perovskite device fabricated by screen‐printing and spin coating with active area of e) 0.05 cm^2^ and f)1.00 cm^2^. g) Long‐term stability of devices by screen‐printing and spin‐coating methods. Reproduced with permission.^[^ ^23^ ^]^ Copyright 2022, Springer Nature.

Very recently, our group developed an ionic liquid methylamine acetate (MAAc) to prepare screen‐printing ink without any thickening agent or crosslinking agent.^[^ ^23^ ^]^ The schematic fabrication of perovskite thin films by screen‐printing method was shown in **Figure** **11**a, and the image of screen‐printing perovskite ink was shown in Figure 11b. It is shown that the viscosity of MAAc was very sensitive to temperature, and it is hundreds of times higher than that of typical organic solvents DMF or Dimethyl sulfoxide (DMSO). The reason for the high viscosity of MAAc was the interaction between ionic liquid anions and cations, while organic solvents have little hydrogen bonds and van der Waals forces. Furthermore, the viscosity of MAAc perovskite ink was investigated at different concentrations. For the 200 , 400, and 800 mg mL^−1^ perovskite ink, the viscosity were in the range of 40–3420, 50–10 100, and 100–44 700 cP respectively. The high viscosity properties of MAAc limited the movement of dissolved perovskites, which can inhibit the deprotonation of MA^+^/FA^+^, thus extending the aging time of screen‐printing perovskite ink. The surface and cross‐sectional SEM of the screen‐printing perovskite films were shown in Figure 11c,d, yielding a dense and pinhole‐free perovskite thin film. viscosity‐dependent morphology and quality of screen‐printed perovskite thin films were investigated. Viscosity had little effect on grain size but significantly affected thickness and roughness of the screen‐printing perovskite film. Since no thickening component was used in perovskite inks, the deposited perovskite film shows better crystal morphology and carrier transport characteristics. It is demonstrated that MAAc‐based perovskite can produce screen‐printed perovskite film with controllable thickness (from ≈120 nm to ≈1200 nm), area (from 0.5 × 0.5 cm^2^ to 5 × 5 cm^2^), and patterning on different substrates. Moreover, printing rates of over 20 cm·s^−1^ and ink utilization close to 100% could be achieved. As shown in Figure 11e,f, using this deposition method in ambient air and regardless of humidity, we obtained the best efficiencies of 20.52% (0.05 cm^2^) and 18.12% (1 cm^2^) compared with 20.13% and 12.52%, for the spin‐coated thin films in normal devices with thermally evaporated metal electrodes, respectively. Importantly, the screen‐printed device retained 92.8% of its original PCE after 4000 h, whereas the spin‐coating device endured a 58.3% PCE loss after 1000 h (Figure 11g).

### 3.6 Counter Electrode

The counter electrodes of current highest‐efficiency n‐i‐p PSCs are usually made of noble metals (e.g, Ag or Au), but the poor stability of the device, which is due to the metal atoms migration into the HTL and continuous degradation at the interface of metal/HTL, is a serious problem.^[^ ^113^ ^]^ To overcome this problem, various low‐cost and widespread carbon‐based CEs with aimed properties have been designed, which are conducive to addressing the shortcomings brought by metal electrodes and promoting important breakthroughs in the commercialization of PSCs. Typically, carbon‐based CEs have excellent thermal stability, good electrical conductivity, and large specific surface area, and screen‐printing is the most widely used method to prepare carbon‐based CEs for PSC modules.^[^ ^114^ ^]^

**Figure 12:**
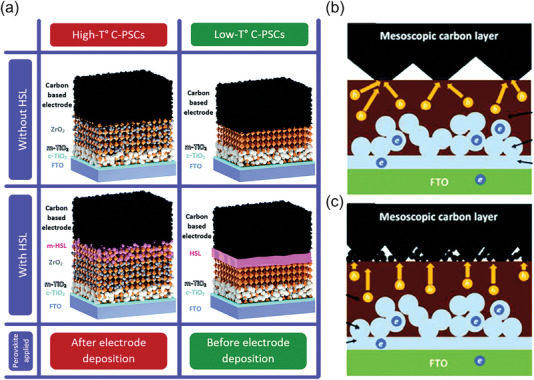
a) Schematic of screen‐printed devices with high‐ and low‐temperature carbon‐based electrodes. Reproduced with permission.^[^ ^123^ ^]^ Copyright 2020, Royal Society of Chemistry. Diagrams of the charge transfer in screen‐printed PSCs based on low‐temperature carbon with b) large graphite flakes and c) small graphite flakes. Reproduced with permission.^[^ ^129^ ^]^ Copyright 2014, Royal Society of Chemistry.

The carbon‐CEs based PSCs (c‐PSCs) have been known as the most competitive choice for the development of cost‐effective, handling, and stable PSCs, owing to the tempting properties of carbon‐based materials, including outstanding stability, low‐cost, water‐repellent, high‐conductivity, and simple transfer process.^[^ ^115^ ^]^ Besides, ion migration in PSCs could be inhibited by carbon materials, naturally to enhance the working stability of PSCs.^[^ ^116^ ^]^ C‐PSCs can be applied in a variety of applications, which requires the carbon paste to exhibit different physical (et. electrical conductivity, thermal conductivity, strength, hardness, elastic modulus, light absorption, refractive index) and chemical properties (et. reactivity, oxidation, adsorption, catalysis). The main raw materials of carbon paste for screen‐printed c‐PSCs include carbon nanotubes,^[^ ^117^, ^118^ ^]^ graphene,^[^ ^119^ ^]^ graphite,^[^ ^120^ ^]^ carbon black,^[^ ^121^ ^]^ and even some natural aloes.^[^ ^122^ ^]^ As shown in **Figure** **12**a, the carbon paste used for screen‐printing can be divided into low‐temperature carbon (LTC) and high‐temperature carbon (HTC), However, the HTC electrode needs a >400 °C annealing process to remove the organic binder, and thus LTC obviously have a broader range of applications since most HTLs and perovskite layers are not resistant to heat. LTC printing ink is mixed with nano‐toner, suitable solvents, and resins/binders, and it should be noted that the annealing temperature of the binder needs to be lower than the decomposition temperature of the perovskite and hole transport materials.^[^ ^123^ ^]^ In binder‐free LTC‐based c‐PSCs, the CEs layer is annealed at a lower temperature (<120 °C) to avoid perovskite degradation. In addition, LTC‐PSCs have more obvious advantages compared with the HTC‐PSCs, such as easy integration with a selective layer, easy control of perovskite crystallization, compatibility with flexible substrates, and higher processing speed. Therefore, researchers mainly focus on optimizing the conductivity, charge selectivity, and perovskite/carbon interface of LTC‐based screen‐printed PSCs.

Liu et al. prepared an LTC printing ink employing different sizes of carbon powder as the carbon source and used hydroxypropyl cellulose as the dispersant, ethyl acetate as the solvent, and polyvinyl acetate (PVAc) as the binder to regulate the viscosity of the ink.^[^ ^124^ ^]^ The CE layer with excellent flexibility and conductivity was fabricated by the low‐temperature process. It is found that the composition of the carbon electrode had a significant impact on the interface contact and device performance of screen‐printed HTL‐free PSCs, where the CE consisting of graphite flake and graphite powder with a weight ratio of 1:2 produces PSCs with PCE of 6.88%. Dileep et al. developed a perovskite printing ink by introducing solid powders, such as hydroxypropyl cell, carbon methylcellulose, graphite flakes, nano graphite powder, and carbon black, into the solvent of polyvinyl acetate (PVAc).^[^ ^125^ ^]^ The conductivity and resistance of printed CE layer were optimized by adjusting the proportion of solid carbon materials, and the CE layer prepared at room temperature (36 ± 1 °C) had high conductivity (49.5 s cm^−1^), low resistance (4.5 Ω cm^−2^) and porous carbon electrodes (45–50 µm). As a result, screen‐printed HTL‐free PSCs based on this low‐temperature carbon electrode achieved PCE of 9.00%. Han et al. developed an inorganic binder to prepare low‐temperature carbon paste, which was prepared by dispersing mixed titanium isopropanol (IV) and acetic acid in terpineol.^[^ ^126^ ^]^ The improvement of viscosity and conductivity of the ink was attributed to ligands interaction between acetic acid molecules and Ti^4+^ to form bidentate bridge and polymerized Ti─O─Ti new species. Duan et al. incorporated boron atoms into the crystal lattice of graphite to improve the work function of the carbon electrode, and the high‐function boron‐doped carbon electrode promoted the hole extraction rate and reduced the charge transfer resistance at the carbon/perovskite interface, ultimately improving the efficiency of the screen‐printed PSCs from 12.84% to 14.04%.^[^ ^127^, ^128^ ^]^ Yang et al. optimized the interface contact between CE and perovskite layer by adjusting the composition and size of graphite and carbon black to improve the carrier transport characteristics and obtained a screen‐printed device with a PCE of 10.2% (Figure 12b,c).^[^ ^129^ ^]^ Currently, through continuous optimization of composition, printing technology, interface contact, and level regulation, the PCE of LTC perovskite devices has exceeded 22%, which is a representative method for low‐cost preparation of high‐efficiency n‐i‐p devices.^[^ ^117^ ^]^ In addition to traditional n‐i‐p or mesoscopic PSCs, the LTC could also be used in some unique PSCs with simplified structures,^[^ ^130^ ^]^ such as carrier transport layer (CTL)‐free or ITO/FTO‐free PSCs. Owing to a work function of −5.0 eV of carbon materials, which is consistent with the energy level of most perovskite materials, LTC is theoretically suitable for CTL‐free devices. Tang et al. developed CTL‐free PSCs with a structure of FTO/CsPbBr_3_/CE, in which graphene quantum dots were used as an interface modifier to further improve the carrier transfer ability.^[^ ^131^ ^]^ Finally, this extremely simple PSCs device obtained PCE of >4%. In 2022, Ma et al. adjusted the Fermi energy level of the perovskite film through additive engineering by introducing 1‐naphthylmethylamine (NMA).^[^ ^132^ ^]^ This n‐type doping formed a favorable n‐p homogeneous junction with the carbon electrode, which effectively promoted the extraction process of holes from the doped perovskite film to the carbon electrode. In addition, NMA could effectively passivate interface defects, reduce non‐radiation recombination, and eventually obtained a PCE of 12.01% in the screen‐printed PSCs with the structure of FTO/MAPbI_3_/carbon. Surprisingly, some works have made efforts to replace the expensive FTO or ITO bottom electrodes by using transparent carbon electrodes, achieving more competitive CTL‐free screen‐printed PSCs with fully carbon electrodes.^[^ ^118^, ^133^ ^]^

### 3.7 Screen‐Printing All Functional Layers

The development goal of perovskite photovoltaic devices is to commercialize with high cost‐effectiveness, which makes fully printing perovskite devices a promising concept.^[^ ^134^, ^135^, ^136^ ^]^ including fully blade coating PSCs,^[^ ^190^ ^]^ fully slot‐die coating PSCs,^[^ ^49^, ^137^, ^138^, ^139^ ^]^ fully inkjet printing PSCs,^[^ ^140^, ^141^ ^]^ and fully spray coating PSCs.^[^ ^142^ ^]^ Currently, for screen‐printing method, the printing of all functional layers has basically been realized except for perovskite film, because the perovskite precursor solution based on organic solvents is not applicable to screen printing method. Alternatively, screen‐printed PSCs have been combined with various methods for preparing perovskite films have been introduced to fabricate screen‐printed PSCs, including slot‐die,^[^ ^143^ ^]^ blade coating,^[^ ^144^ ^]^ inkjet printing,^[^ ^145^ ^]^ spray coating,^[^ ^146^ ^]^ drop casting,^[^ ^147^, ^148^ ^]^ etc. Nevertheless, the realization of fully printed PSCs with screen‐printing of all functional layers is highly desired. Notably, the full‐printing processing scheme based on printing technology has been accepted by the constantly newly constructed perovskite photovoltaic production line. Furthermore, the combination of fully screen‐printing and roll‐to‐roll can improve the industrial competitiveness of perovskite and release the large‐scale potential.^[^ ^149^ ^]^

**Figure 13:**
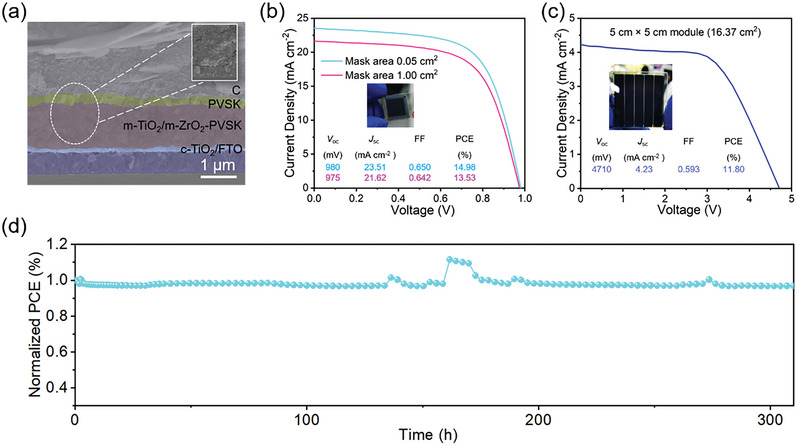
a) Cross‐sectional SEM image of fully screen‐printed PSC modules. b) *J–V* curves of fully screen‐printed perovskite devices with different mask areas. c) *J–V* curves of fully screen‐printed PSC module. d) Operational stability of fully screen‐printed PSCs at maximum power point (0.75 V). Reproduced with permission.^[^ ^23^ ^]^ Copyright 2022, Springer Nature.

Our previous work first developed a screen‐printing perovskite film technique to precisely control the quality of large‐area perovskite film in a point‐line‐plane phase transferring manner.^[^ ^23^ ^]^ As shown in **Figure** **13**a, fully screen‐printed perovskite photovoltaic devices were fabricated by kinetic regulation of screen‐printing films and structure optimization of devices, the advantage of this full printing device was that one machine achieved the preparation of all functional layers including perovskite layer, which significantly reduced the requirement of equipment, technical compatibility and preparation cost. The PCEs of the fully screen‐printed perovskite photovoltaic devices (0.05 cm^2^ and 1.00 cm^2^, Figure 13b) and modules (16.37 cm^2^, Figure 13c) were 14.98%, 13.53%, and 11.80%, respectively. Moreover, the unencapsulated device could maintain 96.75% of its initial efficiency after constant illumination for 300 h at the maximum power point (Figure 13d).

## 4 Strategies for Improving Performance of Screen‐Printed PSCs

A significant advantage of perovskite photovoltaics is solution processing, which is perfectly compatible with high‐throughput roll‐to‐roll (R2R) technology. Currently, for preparing small‐area PSCs, the most commonly used process is spin coating method, which can produce PSCs with a record PCE of 26.08% (certified 25.73%), and such methods can be compatible with various perovskite systems to achieve high efficient PSCs.^[^ ^49^, ^150^, ^151^, ^152^, ^153^ ^]^ However, film‐formation of spin‐coating method is generated by uninterrupted centrifugal force, making it difficult to achieve patterned and scalable thin film deposition. Therefore, to meet the requirements of commercialization, a series of high‐throughput and low‐cost printing methods that are compatible with R2R processes have been developed. At present, the development goals of printing perovskite devices are a larger area (150 cm^2^), higher efficiency (comparable to spin‐coated devices), and excellent stability (IECE 61215:2016).^[^ ^154^ ^]^ Currently, the highest PCE of screen‐printed PSCs exceeds 19%,^[^ ^155^ ^]^ and the device can pass rigorous standard stability testing programs, including the damp heat test (85 °C/85% relative humidity RH), thermal cycling test (−40 °C–85 °C), UV preconditioning test (60 °C, 15 kWh·m^−2^), maximum power point (MPP) tracking light soaking test (55±5 °C,), and even IEC61215:2016 qualification tests with MA‐based perovskite, where the screen‐printed mesoporous PSC device has shown no decay for >9000 h when operating at MPP.^[^ ^156^ ^]^ This inspiring progress is based on the extensity studies that develop effective strategies to improve the device performance of screen‐printed PSCs. In this section, we will summarize the state‐of‐the‐art strategies in this field, including perovskite composition engineering, additive engineering, solvent engineering, and interface engineering.

### 4.1 Perovskite Composition Engineering

**Figure 14:**
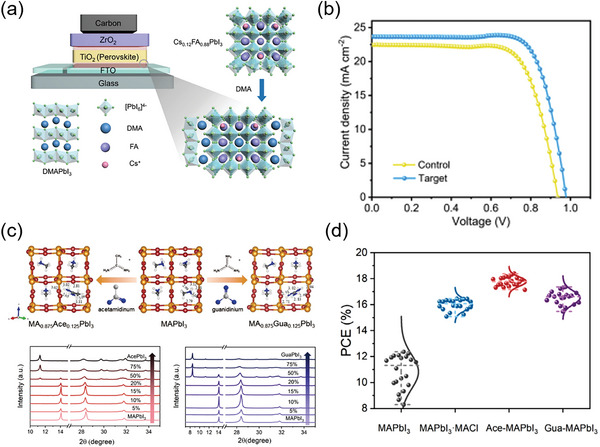
a) Schematic diagram of screen‐printed PSCs based on MAPbI_3_ perovskite with DMA cation doping. b) *J–V* curves of the control and DMA PSCs. Reproduced with permission.^[^ ^158^ ^]^ Copyright 2022, Wiley‐VCH. c) Structure characterizations and XRD patterns of MAPbI_3_ perovskite containing different percentages of Gua cation and Ace cation. d) Box plot of PCE distribution collected from 20 PCSs each. Reproduced with permission.^[^ ^160^ ^]^ Copyright 2021, Elsevier.

According to theoretical calculations, the orbital of A‐site cation has no actual contribution to the energy band edge of perovskite material, but the size and proportion of A‐site cation have obvious significance on the crystal structure and carrier transport characteristics of perovskite material. Hu et al. proved that the cationic FA‐MA mixed perovskite material produced screen‐printed PSCs with improved optoelectronic performance compared to the device based on single A‐site cation perovskite.^[^ ^157^ ^]^ Zhao et al. introduced the dimethylammonium (DMA) cation to partially replace FA sites and form Cs_0.12_FA_0.88‐X_DMA_X_PbI_3_ perovskite film (**Figure** **14**a).^[^ ^158^ ^]^ It is shown that under low‐temperature conditions, a limited quantity of intermediate‐phase DMAPbI_3_ is formed within the perovskite solution, which can act as a template crystal to induce uniform nucleation of the perovskite film. This uniform nucleation facilitates the perovskite solution to adequately fill the mesoporous film, optimize energy level alignment, and ultimately lead to a PCE of 17.46% in screen‐printed PSCs (Figure 14b). Inspired by excessive MAI passivation effect, Wang et al. introduced additional monovalent cations (guanidinium iodide (GUAI), 2‐phenylethylamine hydroiodid (PEAI), and rubidium iodide (RbI)) to improve the performance of screen‐printed PSCs.^[^ ^159^ ^]^ The experimental results show that monovalent cationic halide salt (GUAI, PEAI, and RbI) had a better performance improvement effect than divalent metal halide salt (PbI_2_). Xiao et al. introduce the large cation Ace and Gua into MAPbI_3_ to form hydrogen bonds in the inorganic framework to stabilize the perovskite structure (Figure 14c).^[^ ^160^ ^]^ The hydrogen bonding between GuCl and I could reduce the defect density of perovskite films, resulting in the increase of carrier lifetime and the significant improve of *V*_OC_ of screen‐printed perovskite devices.^[^ ^161^ ^]^ Compared with the original MAPbI_3_, both A‐site macromolecules can significantly enhance the optical absorption and electrical properties of the perovskite film. As shown in Figure 14d, the resultant Ace_0.15_MA_0.85_PbI_3_ perovskite film exhibited excellent carrier mobility and film quality, which greatly increased the PCE of the screen‐printed device to 18.12%. Mei et al. analyzed the main degradation mechanism of MAPbI_3_ in the screen‐printed mesoporous ETL layer and proposed to introduce 5‐AVA iodide into the MAPbI_3_ perovskite to inhibit the crystal decomposition.^[^ ^156^ ^]^ The decomposition of MA perovskite in the mesoporous scaffold is manifested by the escape of MAI in the open space, the reconstruction in the local space, and the irreversible ion migration under external stimulation (light, heat, and electric bias). While the introduction of bifunctional organic molecule 5‐AVA at the perovskite grain boundary could stabilize the MAPbI_3_ crystal, which was conducive to inhibiting the decomposition and promoting the reconstruction of perovskite structure. This strategy produced screen‐printed PSCs with robust stability that passes the standard stability qualification tests of IEC61215:2016. Filonik et al. further studied the effect of 5‐AVAI on the crystallization of perovskite in the mesoporous space.^[^ ^162^ ^]^ Grazing incidence wide‐angle X‐ray scattering (GIWAXS) results showed that 5‐AVAI inhibited the irregular growth of perovskite at the early stage of phase formation, thus avoiding the formation of oversized perovskite grains and promoting the filling of perovskite in the mesoporous ETL. Liu et al. introduced triple cation perovskite Cs_0.05_(FA_0.4_MA_0.6_)_0.95_PbI_2.8_Br_0.2_ into screen‐printed devices with the structure of TiO_2_/Al_2_O_3_/NiO/Carbon for the first time.^[^ ^163^ ^]^ The transient absorption spectra and transient photovoltage/current decay measurements showed that a small amount of Cs increased the diffusion distance and lifetime of perovskite carriers, which was conducive to inhibiting carrier recombination. Based on this technology, screen‐printed devices with PCE of 17.02% were prepared.

In addition to A‐site cation, the doping effect of B‐site cation is also studied. Zhang et al. introduced 10% strontium chloride (SrCl_2_) into MAPbI_3_ perovskite to substantially improve the performance and stability of screen‐printed PSCs.^[^ ^164^ ^]^ Different from the bonding mode of Pb^2+^ and halogen, the coordination number of SrCl_2_ and SrI_2_ was found to be 8 and 7, respectively, which proved that Sr has a highly nondirectional bonding with halogen. This difference indicated that there is competition when incorporating Sr^2+^ into MAPbI_3_, and the experimental results showed that SrCl_2_ can regulate the crystallization process of perovskite and reduce crystal defects. Compared with the control perovskite device without Sr‐doping showing an efficiency of 13%, the PCE of SrCl_2_‐dopted PSCs reached 15.9%. Excess PbI_2_ has been shown to improve *V*_OC_ of screen‐printed PSCs, but it is at the expense of the light stability of the devices.^[^ ^165^ ^]^ In addition, lead‐free perovskite materials, such as (C_6_H_5_NH_3_)BiI_4_, are also been developed in screen‐printed PSCs, which have the potential to prepare low‐cost and stable lead‐free PSCs.^[^ ^166^ ^]^ Zhang et al. prepared high‐performance CsSnI_3_ mesoscopic devices by eliminating Sn^4+^ in precursor solution by electro‐displacement reaction (GDR). Zinc metal powder with reduction potential was introduced into CsSnI_3_ precursor solution to eliminate Sn^4+^.^[^ ^167^ ^]^ At the same time, Zn^2+^ ions were introduced into the perovskite lattice, which was conductive to reduce the adsorption of water and oxygen on CsSnI_3_ surface, thus improving the stability of perovskite materials. Based on this technology, the screen‐printed PSCs with the structure of TiO_2_/Al_2_O_3_/CsSnI_3_/NiO/C achieved PCE of 8.27%.

In addition, the effect of halogen doping in screen‐printed PSCs is also investigated. Chen et al. incorporated F element into MAPbI_3_ perovskite to improve the performance of screen‐printed PSCs.^[^ ^168^ ^]^ Compared to other halogens, F ion has high electronegativity and stronger electron absorption ability, which is conducive to improving the hole transport ability of perovskite materials. Because the tolerance factor t of MAPbI_3_F_3‐X_ perovskite is not within a reasonable range to form a stable perovskite phase, the authors put forward an interesting strategy of replacing I^−^ with BF_4_^−^ that has a similar ionic radius to improve the conductivity of perovskite materials, thus improving the PCE of screen‐printed perovskite device to 13.24%.

### 4.2 Additive Engineering

Exploring coordination additives to control the crystallization process and quality of perovskite films is another effective strategy to improve the performance of screen‐printed PSCs. Ye et al. prepared high‐performance carbon‐based ETL/HTL‐free screen‐printed PSCs by introducing perfluorotetradecanoic acid (PFTeDA) into MAPbI_3_ perovskite.^[^ ^169^ ^]^ The PFTeDA can inhibit harmful ion migration and reduce crystal defects owing to the chelation between the non‐coordinated Pb^2+^ and carbonyl group, and the stability of HTL‐free PSCs was significantly improved with PCE of 18.9%. Wu et al. reported that Biguanide hydrochloride (BH) was able to improve the energy level of perovskite film and promote the preferred growth of perovskite films along (001) and (002) crystal planes.^[^ ^170^ ^]^ The resultant screen‐printed perovskite device showed 16.35% PCE through the introduction of 30% BH. In another work, Yang et al. used 1‐(2‐chlorothal) piperidine hydrochloride (ClEP) as an additive that could passivate non‐coordination ions and defects of perovskite film, which is beneficial to improve *V*_OC_ and PCE of screen‐printed PSCs.^[^ ^171^ ^]^

To better control the crystallization of perovskite, additives with a multi‐coordination effect have been developed. Rong et al. cooperated NH_4_Cl and H_2_O to control the crystallization and growth of perovskite.^[^ ^172^ ^]^ NH_4_Cl was found to retard the crystallization of perovskite, while NH_4_Cl and H_2_O together promoted the formation of intermediate CH_3_NH_3_X·NH_4_PbX_3_(H_2_O)_2_ (X = I/Cl) to promote the preferred orientation of MAPbI_3_ perovskite crystals along^[^ ^109^ ^]^ direction. Accordingly, the screen‐printed PSCs achieved a PCE of 15.6% and 130 days of stability in ambient air with 35% RH. Besides, Yang et al. introduced the multifunctional additive 1‐(2‐chloroethyl) piperidine hydrochloride (ClEP) into the perovskite precursor solution to improve the *V*_OC_ of the device through interface passivation.^[^ ^171^ ^]^ ClEP showed multiple passivation functions for uncoordinated I^−^, vacancy defects, and ion defects during the slow crystallization process. The results show that ClEP plays a significant role in reducing the electron defect density and non‐radiative center of perovskite film. Using this strategy, the PCE of screen‐printed PSCs was increased to 17.08% with excellent stability.

**Figure 15:**
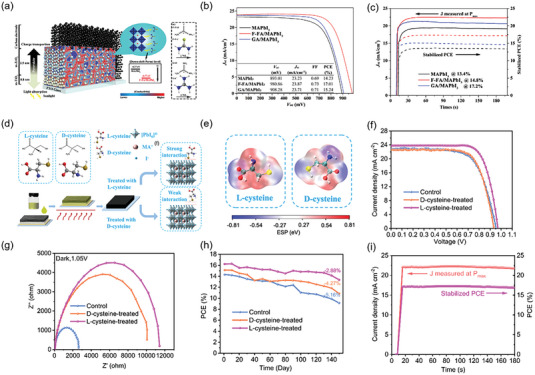
a) Schematic of screen‐printed PSCs with F‐FAPbI_3_ filled inside the mesoporous ETL. b) *J–V* curves and c) steady‐state current density and PCE of the devices of screen‐printed PSCs based MAPbI_3_, F‐FA/MAPbI_3_, and GA/MAPbI_3_ films. Reproduced with permission.^[^ ^173^ ^]^ Copyright 2021, Wiley‐VCH. d) Screen‐printed devices based on the additive of l‐cysteine and d‐cysteine. e) Calculated electrostatic potential (ESP) profiles of d‐cysteine and l‐cysteine. f) *J–V* curves, (g) Nyquist plots, and h) stability test of the champion devices without, with d‐cysteine and l‐cysteine treatment. i) Steady‐state outputs of current density and PCE of the screen‐printed PSCs with l‐cysteine treatment. Reproduced with permission.^[^ ^181^ ^]^ Copyright 2022, Wiley‐VCH.

Liu et al. substitute the amino group on guanidinium (GA^+^) with F group to prepare the additive *N*, 1‐fluoroformamidine iodide (F‐FAI).^[^ ^173^ ^]^ As shown in **Figure** **15**a, F‐FA has stronger molecular polarity due to the electron‐withdrawing effect of F compared to the additive GA^+^, which can effectively improve the crystal quality of perovskite films and optimize the Fermi‐ energy level. Screen‐printed PSCs based on F‐FAI showed significantly improved PCE from 15.24% (GA^+^) to 17.01% (Figure 15b). In Figure 15c, the stabilized PCE of MAPbI_3_, GA/MAPbI_3_, and F‐FA/MAPbI_3_ devices were 13.4%, 14.8%, and 17.2%, respectively. Similarly, lewis base 2‐(methylthio) ethylamine hydrochloride (MTEACl) can interact with un‐coordinated Pb^2+^ as an electron donor, and sulfur donor can effectively passivate film defects and improve device performance.^[^ ^174^ ^]^ Ko et al. found that the introduction of thiourea into perovskite solution significantly affected the growth process of perovskite crystals and the performance of screen‐printed devices.^[^ ^175^ ^]^ Thiourea can form hydrogen bonds and lewis acid‐base adducts with I and Pb atoms, respectively, which contributes to retarding the growth of perovskite crystals, suppressing the formation of superoxide anion radicals and I_2_, and promoting the formation of dense crystals in mesoporous ETL. Therefore, the light absorption characteristics and the carrier mobility of the screen‐printed perovskite film using thiourea additive were enhanced, and the perovskite device showed PCE of 13.03% with excellent stability. Chen et al. developed an “automatic dual‐site passivation” strategy to prepare high‐performance screen‐printed PSCs.^[^ ^176^ ^]^ The strategy was realized by introducing the tautomer of imidazole anions, 1‐allyl‐3‐methylimidazole tetrafluoroborate ([AMIm]BF_4_) and 1‐benzyl‐3‐methylimidazole tetrafluoroborate([BzMIm]BF_4_) with different side chains as additive, which can jointly passivate perovskite film. Specifically, the lone pair electrons of N in imidazole directly passivate both Pb ions and the side chain methylene iodide ions due to the inhibition of MA migration by the imidazole ring. Moreover, [AMIm]BF_4_ and [BzMIm]BF_4_ can also inhibit the non‐radiative recombination of perovskite film and improve the interface carrier mobility of screen‐printed devices. Based on this strategy, a screen‐printed perovskite device achieved a high PCE of 16.87%. Yang et al. introduced the additive octyltrimethylammonium chloride (OTAC) into MAPbI_3_ to optimize the energy level alignment and filling capacity.^[^ ^177^ ^]^ Liu et al. modified MAPbI_3_ with Cesium acetate (CsAc) and realized the control of perovskite energy level alignment at the perovskite/carbon interface.^[^ ^178^ ^]^ MA_1‐X_Cs_X_PbI_3_ on the surface layer was produced from CsAc with MAPbI_3_, which could effectively reduce the recombination of carriers and improve the film quality. The screen‐printed device based on CsAc post‐processing has almost no hysteresis and no performance degradation for 4 months. In the process of two‐step screen‐printed perovskite device preparation, the concentration and soaking time of perovskite solution are critical. In addition, Alon et al. observed the process of device performance recovery under dark conditions, which may be due to the stress release in the recrystallization process caused by the internal shrinkage of perovskite, helping to improve the device stability.^[^ ^179^ ^]^ In addition, Du et al. produced an I‐rich region and Pb‐rich region by gradient self‐doping method in the two‐step deposition method, resulting in the work function difference of perovskite in different layers, which promoted the directional transmission of carriers and improved the PCE of screen‐printed perovskite devices to 17.68%.^[^ ^180^ ^]^

Wu et al. introduced l‐ and d‐cysteine as additives to explore the effect of chiral molecular environment on the performance of screen‐printed PSCs.^[^ ^181^ ^]^ Figure 15d shows the molecular structure formula of l/d‐cysteine and the preparation process of screen‐printed PCSs. As shown in Figure 15e, the chiral molecular environment of l‐cysteine accelerates the intramolecular charge transferring, resulting in a negative surface electrostatic potential around the carboxyl group that significantly passivates the unpaired Pb^2+^. As shown in Figure 15f, the PCE of screen‐printed PSCs based on d‐cysteine, and l‐cysteine additives reached 15.12 and 17.41%, respectively. As shown in Figure 15g, the EIS curves showed that the order of R_rec_ in different devices is control<d‐cysteine‐treated<l‐cysteine‐treated, indicating l‐cysteine‐treated has a higher suppression capability of charge recombination. As shown in Figure 15h, the long‐term stability showed the l‐cysteine‐treated devices enhanced the protective capacity for screen‐printed devices owing to excellent hydrophobicity. The best‐performing device with l‐cysteine treatment showed a steady‐state current density of 22.28 mA cm^−2^ and a stabilized PCE of 17.04% at 0.765 V (Figure 15i). For Pb‐free CsSnI_3_‐based devices, Ban et al. adjusted the balance between the nucleation and growth of CsSnI_3_ perovskite crystals by two‐step thermal annealing and Sn^2+^ coordination with 1‐(4‐carboxyphenyl)‐2‐thiourea additive and the screen‐printed perovskite device could reach PCE of >8%.^[^ ^182^ ^]^

### 4.3 Solvent Engineering

**Figure 16:**
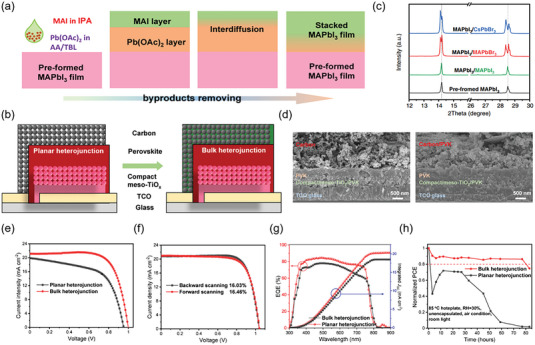
a) Planar heterojunction screen‐printed PSCs. b) XRD spectra of the perovskite/perovskite stacking films. c) Planar heterojunction and bulk heterojunction (BHJ) C‐PSCs based on a two‐step orthogonal solvent method to stack the perovskite films. d) Cross‐sectional SEM images of planar heterojunction and BHJ. e) *J–V* curves of the screen‐printed PSCs for planar and bulk heterojunctions. f) *J–V* curves of the champion screen‐printed PSCs with the BHJ. g) external quantum efficiency (EQE) spectrum and integrated current density for planar and bulk heterojunctions. h) Thermal stability of the unencapsulated screen‐printed PSCs. Reproduced with permission.^[^ ^187^ ^]^ Copyright 2022, Wiley‐VCH.

For solution processing of perovskite ink in screen‐printing technique, solvent engineering also plays a key role. Ming et al. found that a mixed solvent of ethanol (EtOH) and γ‐butyrolactone (GBL) can effectively inhibit the formation of perovskite seed crystals.^[^ ^183^ ^]^ Specifically, EtOH can effectively improve the solubility of PbI_2_ and weaken the coordination between solvent molecules and Pb^2+^ ions, which promotes the crystallization and infiltration of perovskite in the screen‐printed mesoporous layer. Wang et al. used *N*‐methylformamide (NMF) solvent to prepare CsPbBr_3_ perovskite films in screen‐printed PSCs and found that NMF showed better crystallization orientation along the (100) and (110) crystal direction with improved film quality compared to DMF/DMSO mixed solvent.^[^ ^184^ ^]^ Therefore, an HTL‐free screen‐printed perovskite device achieved a PCE of 8.32%. Cheng et al. introduced green solvent ethyl acetate (EA) and CsBr to improve the performance of MAPbI_3_‐based screen‐printed PSCs.^[^ ^185^ ^]^ In this system, EA was used as an anti‐solvent and CsBr was used as an additive to regulate the crystal crystallization. The synergistic effect of CsBr and EA improves the quality of MAPbI_3_ perovskite films and reduces their density of defect states, thus improving the performance of PSCs with PCE of 16.45%. Liu et al. prepared screen‐printed PSCs at room temperature using the mixed solvent composed of methylamine ether solution (MA‐EtOH solution) and acetonitrile (ACN).^[^ ^186^ ^]^ In the mixed solvent, ink can fully fill in the mesoporous layer and the perovskite crystals are rapidly crystallized at room temperature, providing a method for preparing screen‐printed PSCs with PCE of 15.03% and low energy consumption. Liu et al. developed a two‐step orthogonal solvent method to deposit a series of perovskite films with different components on the prefabricated MAPbI_3_ film and controlled the composition and band alignment of perovskite in a low‐cost way.^[^ ^187^ ^]^ As shown is **Figure** **16**a, the carrier extraction of the conventional planar heterojunction was limited due to poor perovskite/carbon interface contact. To improve interface contact, a perovskite/carbon bulk heterojunction (BHJ) was fabricated by dropping another perovskite precursor into the carbon material (Figure 16b). Specifically, the solvent system was made up of a mixture of acetic acid (HOAc) and tertbutyl alcohol (TBA) for lead acetate (Pb(OAc)_2_), and iso‐propanol (IPA) for MAI. This solvent system allows for the deposition of the free perovskite onto the preformed perovskite film while maintaining the integrity of the original layer. Once the Pb(OAc)_2_/MAI composite layer is deposited onto the preformed perovskite layer, the free perovskite film is created by heating and removing MAOAc byproducts. As shown in Figure 16c, MAPbI_3_/MAPbI_3_ shows no change in the original crystal orientation, while the XRD patterns show two main peaks at 14.2° for the stacked perovskite with MAPbBr_3_/MAPbI_3_ and CsPbBr_3_/MAPbI_3_ films on the preformed MAPbI_3_. Figure 16d shows the cross‐sectional SEM images of planar heterojunction and BHJ in a screen‐printed device. After the introduction of free perovskite on the surface of the carbon electrode, the PCE of BHJ PSCs increased to 15.23% from 11.57% of the planar heterojunction PSCs (Figure 16e), and the PCE of the optimal device was >16% in both forward and backward scanning (Figure 16f). Figure 16g shows the integrated *J_SC_* of the planar junction and BHJ screen‐printed PSCs are 18.50 and 20.33 mA cm^−2^ respectively, which are consistent with values of *J–V* measurement. As shown in Figure 16h, after aging at 85 °C/30%RH for 80 h, the unencapsulated screen‐printed PSCs with planar heterojunction almost fail, while BHJ devices still retain 80% of the initial PCE.

**Figure 17:**
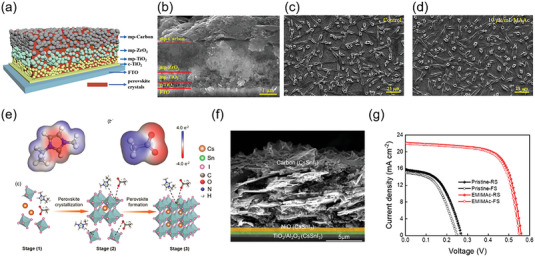
a) Schematic diagram, b) cross‐sectional SEM image, and c,d) surface SEM images of screen‐printed PSCs based on ionic liquid MAAc as co‐solvent. Reproduced with permission.^[^ ^192^ ^]^ Copyright 2022, American Chemical Society. e) Surface electrostatic potential distribution and the role of EMIMAc in the formation of CsSnI_3_. f) The cross‐sectional SEM image of screen‐printed CsSnI_3_ perovskite device with the structure of TiO_2_/Al_2_O_3_/NiO/carbon. g) *J–V* curves of the pristine and EMIMAc‐treated screen‐printed PSCs. Reproduced with permission.^[^ ^194^ ^]^ Copyright 2022, Wiley‐VCH.

In addition to conventional organic solvents, novel ionic liquids have also been developed as solvents, which can regulate the crystallization process of perovskites through interface modification, energy alignment, or defect passivation.^[^ ^188^, ^189^, ^190^, ^191^ ^]^ Wang et al. introduced the ionic liquid methylamine acetate (MAAc) as a co‐solvent into MAPbI_3_ precursor solution to regulate the crystallization process and pore filling of perovskite in mesoporous ETL (**Figure** **17**a,b),^[^ ^192^ ^]^ Due to the strong Pb−O interaction between MAAc and PbI_2_ and the formation of N−H···I hydrogen bond, the mesophase of MAPbI_3−X_(Ac)_X_ will form, which effectively retards the crystallization rate of perovskite phase. As shown in Figure 17c, the pristine perovskite films contain irregular holes and rod/textile‐like structures, indicating that perovskite does not adequately cover the surface of the mesoporous ETL. In contrast, the perovskite films with MAAc co‐solvent exhibit uniform crystal morphology and adequate coverage (Figure 17d), which is attributed to the retarded crystallization of perovskite in the presence of MAAc. As a result, PCE of MAAc‐based screen‐printed PSCs was increased from 10.90% to 13.54%. Similarly, ionic liquid 1‐ethylpyridine chloride (1‐EC) could accelerate the nucleation and retard the growth rate of perovskite crystals, and an SEM image shows that perovskite crystals are fully filled in the mesoporous layer.^[^ ^193^ ^]^ By introducing ionic liquid EMIMAc, Ban et al. controlled the deep‐level defects in CsSnI_3_ perovskite films, which were induced by unpaired Sn^2+^ ions.^[^ ^194^ ^]^ As shown in Figure 17e, EMIMAc has strong electrical coordination with Sn^2+^ on the surface of perovskite, which originates from the lone electron pair of carboxyl functional group in EMIMAc and the π electron donor from the electron‐rich imidazole part. This coordination effect is beneficial for increasing the formation energy of uncoordinated Sn^2+^ and reducing the density of defect, so the corresponding non‐radiative recombination is effectively suppressed and the carrier lifetime is prolonged. CsSnI_3_ PSCs based on screen‐printing technology with a structure of c‐TiO_2_(50 nm)/m‐TiO_2_(400 nm)/Al_2_O_3_ (450 nm) /NiO (800 nm)/ thick carbon (10 µm) (Figure 17f) shows PCE of 8.54% PCE with *V*_OC_ of 0.56 V (Figure 17g).

Interestingly, Guan et al. developed a solvent‐free in‐situ crystal transfer (ICT) strategy to prepare screen‐printed PSCs with a high filling rate.^[^ ^195^ ^]^ The process is as follows: The solid MAPbI_3_ single crystal was first placed in the MA^0^ gas stream to convert it into liquid phase and filled in the mesoporous support, which was then transferred to the N_2_ stream to promote the formation of high‐quality solid perovskite nanocrystals again. Compared with the traditional drop‐casting method, the perovskite thin films prepared by ICT method show the interconnected morphology, which can effectively improve the lifetime of photogenerated carriers from 37.52 ns to 110.85 ns. Finally, ICT‐based screen‐printed PSCs show a PCE of 15.89%.

### 4.4 Interface Engineering

There are many functional layers involved in the screen‐printed PSCs, and the quality of the interfaces greatly determines the performance of the device.^[^ ^196^ ^]^ Therefore, interface engineering has been developed to optimize the device.^[^ ^197^, ^198^, ^199^, ^200^, ^201^ ^]^

**Figure 18:**
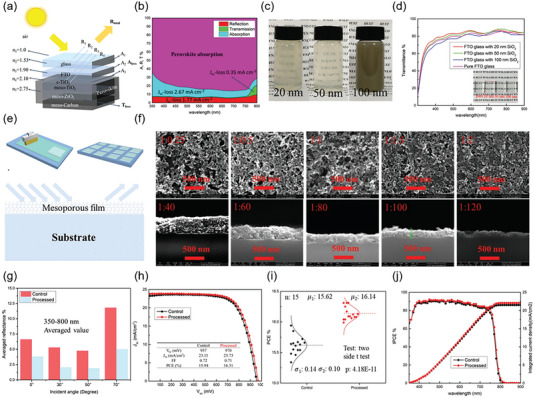
a) Optical loss analysis and b) optical losses and current density losses for screen‐printed PSCs. c) Photograph of SiO_2_ screen‐printing slurries with different particle sizes. d) Transmittance curves of FTO glass without and with screen‐printing SiO_2_ films. e) Schematic of screen‐printing m‐SiO_2_. f) SEM images of m‐SiO_2_ films with different mass ratios of SiO_2_/ethyl cellulose and the cross‐sectional SEM images of m‐SiO_2_ films with different thicknesses. g) Average reflectance values of control and processed with different incident angles. h) *J–V* curves of champion devices. i) PCE distribution of m‐SiO_2_ screen‐printed PSCs. j) IPCE and integrated current densities of control and processed devices. Reproduced with permission.^[^ ^205^ ^]^ Copyright 2022, Wiley‐VCH.

For the interface of transparent back electrode, Ye et al. modified ITO paste by mixing ITO powder, ethyl cellulose, and terpineol, which was used to screen‐print the back ITO electrode.^[^ ^202^ ^]^ The authors screen‐printed TiO_2_, ZrO_2,_ and ITO layer by layer to prepare an all‐inorganic mesoporous scaffold. By optimizing the mesoporous particle size, dispersant, and annealing temperature, mesoporous ITO (m‐ITO) films with mobility of 7.5 cm^2^V^−1^s^−1^ and visible light transmittance (AVT) of over 80% were prepared. Using this technology, 9.85% PCE of semi‐transparent screen‐printed device and 13.92% PCE of four‐terminal perovskite/silicon tandem device with excellent stability were successfully prepared. Sohmer et al. proposed a method of screen‐printing m‐ITO, which was applied to HTL‐free PSCs.^[^ ^203^ ^]^ The advantage of this screen‐printed device is that the transparent contact allows the use of this structure in double‐sided configurations requiring additional layers. Finally, the screen‐printed device showed 18.3% efficiency based on a double‐sided configuration. Jiang et al. tried to prepare a simple device structure without BL and HTL and prepared an HTL‐free screen‐printed device with a conversion efficiency comparable to the presence of c‐TiO_2_.^[^ ^204^ ^]^ Because the wettability of perovskite solution on TiO_2_ is significantly better than that on FTO, the poor contact between perovskite and FTO was caused, which was conducive to inhibiting the carrier recombination at the interface of FTO/perovskite. This simplified screen‐printed device finally produced a PCE exceeding 13%. Wang et al. prepared the m‐SiO_2_ antireflective coating on the glass side of the FTO conductive substrate by screen‐printing method to reduce the optical loss of the air/glass interface.^[^ ^205^ ^]^ As shown in **Figure** **18**a, the optical losses for screen‐printed PSCs include the air/glass interface (*R*_1_), glass/FTO interface loss (*R*_2_), the FTO/compact TiO_2_ interface loss (*R*_3_), and the compact TiO_2_/perovskite interface (*R*_4_). As shown in Figure 18b, the *R*_1_, *R*_2_, *R*_3,_ and R_4_ were 4.39%, 1.16%, 0.28%, and ≈0% at 550 nm according to the Fresnel equation, and thus the sum of *R*_1_, *R*_2_, *R*_3_, and *R*_4_ was ≈5.83% and led to the current density loss (*J_loss_*). As shown in Figure 18c, m‐SiO_2_ films with different nanoparticles were used as anti‐reflection coatings to achieve the desired refractive index. The authors screen‐printed m‐SiO_2_ onto the FTO glass and measured the transmittance of those samples, the transmittance was significantly enhanced for 20 nm m‐SiO_2_ sample (Figure 18d,e). They further studied the influence of the porosity and thickness on the transmittance of m‐SiO_2_ film, which was 85% to 89% by screen‐printed m‐SiO_2_ antireflection with gradient index interface (Figure 18f). Figure 18g shows that the m‐SiO_2_ has the ability to reduce the reflection of the sunlight incident with any angle. When the incidence Angle reached 70°, the average reflectance was greatly reduced by 6.81%. Finally, the PCE of screen‐printed PSCs was enhanced to 16.31% from 15.94% owing to improved *J_SC_* (Figure 18h), and the PCE distribution confirmed the improvement of screen‐printed PSCs (Figure 18i). As shown in Figure 18j, the integrated *J_SC_* is enhanced from 21.51 mA cm^−2^ to 22.11 mA cm^−2^, which is consistent with the result of device performance.

**Figure 19:**
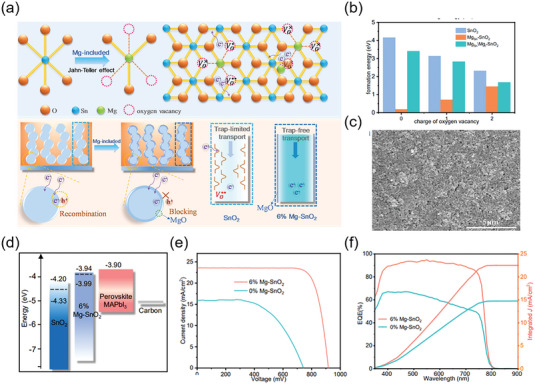
a) Illustration of self‐doping, charge recombination, and transport influenced by Mg. b) Formation energies of OVs calculated by DFT. c) SEM images of the m‐SnO_2_ introduced 6%Mg. d) Energy level alignment, e) *J–V* curves, and f) EQE of screen‐printed PSCs based on 0% and 6% Mg─SnO_2_. Reproduced with permission.^[^ ^207^ ^]^ Copyright 2022, Wiley‐VCH.

For ETL/perovskite interface, Lie et al. introduced a layer of self‐assembled organic silane between the m‐TiO_2_ and the perovskite layer to realize the interface control of the screen‐printed device, which could optimize the energy level alignment of the device and enhance the carrier lifetime of perovskite layer.^[^ ^206^ ^]^ Organic silane‐based screen‐printed PSCs showed PCE of 12.7%, which was one of the early cases of interface control methods in screen‐printed devices. Liu et al. reported preparation of magnesium (Mg) di‐l‐aspartate doped m‐SnO_2_ screen‐printed paste for preparing high‐performance screen‐printed perovskite devices.^[^ ^207^ ^]^ As shown in **Figure** **19**a, the formation of oxygen vacancies (OVs) and SnO_2_ self‐doping process are managed by the synergism of lattice Mg and interstitial Mg. Due to the difference in coordination number between Sn^4+^ and Mg^2+^ and oxygen, lattice Mg (Mg_Sn_) tends to make V_0_^×^ non‐contributing electrons, and the cooperation of Mg_i_ and V_0_^×^ promotes the generation of n‐doped electrons in SnO_2_. Figure 19b shows that Mg_Sn_─SnO_2_ had a smaller formation energy, indicating the Mg could introduce OVs into SnO_2_. Figure 19c shows the surface morphology of 6% Mg─SnO_2_ mesoporous film, and this mesoporous film based on OVs management method is beneficial for improving the carrier mobility and Fermi energy level of SnO_2_ (Figure 19d). As a result, the screen‐printed Mg‐doped m‐SnO_2_ ETL could reduce the defect density of perovskite film and inhibit the interface recombination, producing PSCs with PCE exceeds 17% (Figure 19e). As shown in Figure 19f, the higher EQE and integrated current density demonstrated the improved charge collection of 6% Mg─SnO_2_.

Mesoporous materials have been regarded as an indispensable part of screen‐printed devices, and they can promote the carrier extraction process of perovskite nanocrystals and greatly avoid carrier recombination caused by hole accumulation.^[^ ^208^ ^]^ He et al. fabricated an extremely thin ZrO_2_ film on the surface of m‐TiO_2_ to form an embedded structure.^[^ ^209^ ^]^ The Zr^4+^ can be inserted into the lattice of TiO_2_ and works with Ti^4+^and O^2−^, reducing the oxygen vacancy and defect state density on the surface of the screen‐printing TiO_2_ film, which effectively inhibited the carrier recombination at the TiO_2_‐perovskite interface and improves the carrier transport behavior. As a result, the screen‐printed device of HTL‐free with composition of Cs_0.05_(FA_0.92_MA_0.08_)_0.95_Pb(I_0.92_Br_0.08_)_3_ obtained a PCE of 17.81%. Lanthanide materials as down‐conversion materials, such as lanthanide‐containing polyoxometalate Na9[EuW10O36], could convert high‐energy photons into low‐energy visible photons to improve the utilization of visible light by perovskite materials.^[^ ^210^ ^]^ As a result, the m‐TiO_2_ doped by EuW10O36 shows improved *J_SC_* and PCE of screen‐printed perovskite devices. Li et al. developed a novel m‐TiO_2_ with cake structure, high specific surface area, and excellent wettability at 500 °C by sintering metal‐organic framework (MOF) materials NH_2_‐MIL‐125.^[^ ^211^ ^]^ The NH_2_‐MIL‐125 has better permeability and crystallinity to perovskite solution than traditional m‐TiO_2_, so the device performance of screen‐printing is significantly improved. Recently, Lin et al. developed a quasi‐interdigitated finger‐shaped back electrode containing an m‐TiO_2_ charge‐transferring layer, which was used to improve the carrier mobility and collection stability at the m‐TiO_2_/perovskite Schottky interface, and had an important value for the performance improvement of screen‐printed perovskite devices.^[^ ^212^ ^]^ Wang et al. used aluminum and indium co‐doped TiO_2_ (AlIn‐TiO_2_) as a dense layer to optimize the performance of screen‐printed perovskite devices.^[^ ^213^ ^]^ Compared with 13.49% PCE of control device, AlIn‐TiO_2_ device showed a 15.53% PCE, which came from more reasonable band alignment and convenient carrier transport behavior. Liu et al. synthesized barium stannate (BaSnO_3_) nanocrystals by adjusting the crystallization process and oxygen vacancy distribution.^[^ ^214^ ^]^ Compared with oxygen, nitrogen atmosphere helps to improve the crystallinity and oxygen vacancy of BaSnO_3_, which is conductive to improve the uniformity and dispersion of BaSnO_3_. Finally, screen‐printed perovskite devices based on BaSnO_3_ show 14.77% PCE. Liu et al. found that Eu^3+^ doping could improve energy‐level alignment and crystal quality in CsPbBr_3_ lattice, enhancing photoabsorption and current density.^[^ ^215^ ^]^ Xu et al. loaded N719 on TiO_2_ surface by anchoring carboxyl group.^[^ ^216^ ^]^ Taking TiO_2_ nanoparticles as the core promotes the crystallization of perovskite, and N719 with carboxylic acid in any direction could enhance the passivation of MA vacancies on the surface, especially for irregular perovskite crystals in mesoporous materials. Finally, the device performance was significantly improved due to the passivation effect of MA vacancy and the charge extraction process, and the *J_SC_* was naturally improved.

**Figure 20:**
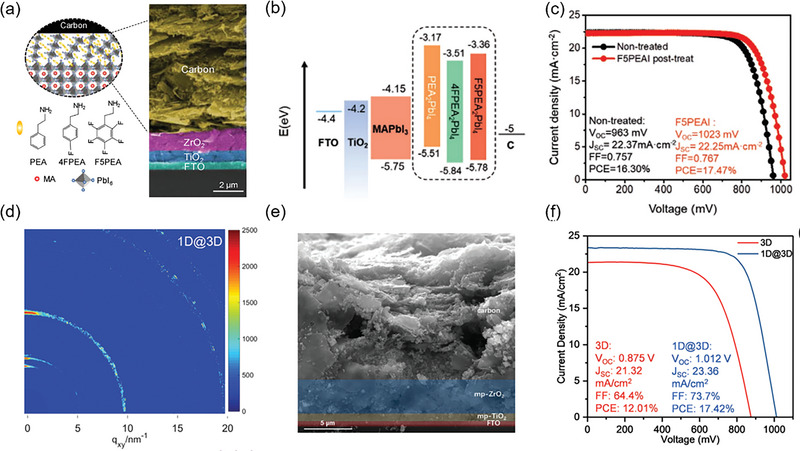
a) Schematic of screen‐printed PSCs with PEAI, 4FPEAI and F5PEAI post‐treatment. b) Energy level alignment diagram of screen‐printed PSCs with a 2D electron blocking layer at MAPbI_3_/carbon interface. c) *J–V* curves of the screen‐printed PSCs with and without F5PEAI post‐treatment. Reproduced with permission.^[^ ^227^ ^]^ Copyright 2021, Wiley‐VCH. d) GIWAXS of 1D@3D perovskite films prepared on m‐TiO_2_. e) Cross‐sectional SEM of the device with a structure of glass/FTO/c‐TiO_2_/m‐TiO_2_/m‐ZrO_2_/carbon. f) *J–V* curves of the optimized 3D and 1D@3D PSCs. Reproduced with permission.^[^ ^228^ ^]^ Copyright 2022, Wiley‐VCH.

For perovskite/HTL interface, Xia et al. adjusted the energy level alignment at the perovskite/carbon interface by using the P‐doping of F4TCNQ molecule.^[^ ^217^ ^]^ The upward energy band bending is conducive to hole extraction and reducing charge recombination, and the screen‐printed perovskite device with an HTL‐free structure can provide >18% PCE. Similarly, Zhang et al. introduced 2‐Bromo‐6‐fluoronaphthalene (BFN) to adjust the interface energy level of perovskite film.^[^ ^218^ ^]^ The terminal electrophilic point of BFN interacts with the iodine ion in Cs‐FA‐MA perovskite to form a non‐covalent halogen bond. At the same time, the conjugated fusion skeleton of naphthalene helped to attract electrons from perovskite layer. The double effect of BFN attracting electrons contributed to the movement of perovskite CBM, VBM, and *E*_F_ and make the better alignment of energy levels at the interface, which accelerates the hole mobility at the perovskite/carbon interface. Accordingly, the carrier loss at the interface is effectively suppressed, and thus the screen‐printed perovskite device based on post‐treatment with BFN shows the best performance of 16.77% PCE. Yang et al. designed a polyethylene imine functionalized carbon nanotube (PEI/CNT) interlayer as a dual‐functional interface bridge to prepare a fully printing all‐inorganic perovskite device.^[^ ^219^ ^]^ The Cl‐modified graphite (prepared by mixing graphite powder, HCl solution, and NaClO solution) has been proven to improve the PCE of screen‐printed devices.^[^ ^220^ ^]^ The functionalized PEI/CNT interlayer could connect the upper and lower layers to form a high‐quality perovskite/carbon interface due to the molecular coordination ability. The results show that PEI/CNT molecules reduce the interfacial transfer resistance and passivate the surface defects of perovskite films. The PCE of the prepared all‐printing all‐inorganic C‐PSC was 10.55% with an FF of 0.77, while the PCE of the device without PEI/CNT bridge was only 7.41% with an FF of 0.56. In addition, the lamination process was also found to improve the carrier transport ability of carbon materials, enhancing the performance and reliability of devices.^[^ ^221^ ^]^ Reddy et al. introduced a series of D‐π‐D porphyrin molecules (HPPHT, CPPHT, and ZPPHT) between nanoparticle‐graphene composite and perovskite, and replaced the target energy level to the surface of perovskite through low‐temperature molecular engineering process to improve the performance of printing devices.^[^ ^222^ ^]^ CPPHT systems have lower activation energy and better electrical properties than the other two systems. Moreover, due to shorter bond lengths and more obvious π–π stacking, CPPHT‐based screen‐printed perovskite devices showed the highest PCE. In another work, vanadium oxide (VO_X_) was treated with the interface of carbon/perovskite to improve the energy level alignment of screen‐printed devices.^[^ ^223^ ^]^ Generally, non‐photoactive δ‐FAPbI_3_ is considered an obstacle to efficient perovskite devices, and Chen et al. found that mixed α/δ‐FAPbI_3_ existed at the perovskite/carbon interface in high humidity environment at the surface of FAPbI_3_, which is conducive to the energy band alignment of the device and the reduction of carrier recombination at the interface.^[^ ^224^ ^]^ In addition, Micro volume expansion caused by the phase transition from α‐FAPbI_3_ to δ‐FAPbI_3_ reduced the contact resistance at the interface and significantly improved the *V_OC_* and FF of screen‐printed perovskite devices, and obtain 17.11% PCE. Liu et al. reported that cellulose‐based activated carbon (CAC) optimized the energy level matching of screen‐printed devices based on CE.^[^ ^225^ ^]^ The optimized carbon film had high oxygen content and a large specific surface area, which contributed to the accelerated carrier mobility and the improved performance of screen‐printed devices. Xiao et al. used 3‐chlorothiophene (3‐CT) and 3‐thiophene ethylenediamine (3‐TEA) to passivate the defects on screen‐printed perovskite film.^[^ ^226^ ^]^ 3‐TEA with larger molecule sizes could form 2D perovskite films that block the reverse transmission of electrons, while 3‐CT could passivate the perovskite defects in the entire mesoporous support. This post‐processing strategy effectively improves the PCE of screen‐printed devices to 18.49%. Similarly, the pentafluorphenylethylammonium iodide (F5PEAI) was used to reduce the dimension of the perovskite surface, forming type‐II band alignment by F5PEA_2_MA_n‐1_PbnI_3n+1_ at the carbon electrode/perovskite interface (**Figure** **20**a,b).^[^ ^227^ ^]^ These 2D/3D perovskite heterostructures are conducive to improving the photovoltage of screen‐printed perovskite devices and achieved a high PCE of 17.47% (Figure 20c). Wu et al. grew a layer of 1D TFPbI_3_ on the surface of 3D perovskite film using thinififormamine hydrochloride (TFCl).^[^ ^228^ ^]^ As shown in Figure 20d, low‐dimensional TFPbI_3_ passivates the defects of perovskite and induces the directionally perpendicular growth of perovskite on the substrate, which significantly improves the carrier mobility and inhibited harmful carrier recombination. Figure 20e shows the cross‐sectional SEM of the 1D/3D screen‐printed perovskite devices, and the devices show a high efficiency (17.42%) with excellent stability (Figure 20f).

## 5 Summary and Outlook

### 5.1 Summary

In this review, we have given a critical overview of the advanced development of PSCs fabricated by screen‐printing technology. We started with the introduction of the theoretical models and key parameters of screen‐printing technology and compared the features of screen‐printing method with other printing methods. Then, functional layers in PSCs deposited by screen‐printing method were summarized, including hole blocking layer, electron transport layer, insulating layer, hole‐transporting layer, perovskite layer, and counter electrode, based on which the latest developments of fully screen‐printed PSCs were discussed. Furthermore, the state‐of‐the‐art strategies that optimize the device performance of screen‐printed PSCs were systematically reviewed. This review highlights the significance of developing low‐cost, efficient, and large‐scale PSCs based on screen‐printing technology, which opens up new avenues for promoting the practical commercialization of PSCs.

### 5.2 Outlook

With up to 26.1% of PCE, third‐generation PSCs are highly competitive in the photovoltaic field. In the last few years, with the goal of replacing existing photovoltaic technologies with low energy costs and simple processing processes, it already has a clear technical path and development plan for the commercialization of PSCs. Nevertheless, in order to be widely accepted by the industry, there are still two major challenges to be solved for PSCs: stability and cost. Screen‐printing technology has been proved to be a reliable solution for the production of efficient PSCs with low‐cost and large‐scale, and the realization of fully screen‐printed PSCs could greatly promote the industrialization of PSCs. However, there are still several immediate challenges to be urgently solved in the commercialization process of fully screen‐printed PSCs.

First, further improving the PCE of the screen‐printed PSCs. The device performance is still relatively low owing to the uncontrolled crystallization process of perovskite in the mesoporous interior and the obstruction of carrier transport at the perovskite/carbon interface. The ink characteristics (surface area, particle size, porosity, pore size) and electrical characteristics (conductivity, carrier diffusion lengths) of the mesoporous material should be carefully optimized, which could improve the crystallinity and crystal orientation of perovskite. In addition, the energy‐level alignment between CE and perovskite deserves further study. In particular, the composition of carbon materials and the interface modification of perovskite still have the potential to significantly improve device performance, because the improved interface bind banding can improve carrier extraction and collection.

Second, prolonging the operational stability of the screen‐printed PSCs. The screen‐printed PSCs with a porous structure can offer improved resistance to adverse environmental factors such as humidity, heat, and UV rays, achieving long‐term light stability for thousands of hours. However, it is still difficult to compete with current silicon solar cells. The decomposition of perovskite in the face of water and oxygen and insufficient crystallization are the main factors leading to the device failure. In order to further improve the stability of screen‐printed PSCs, continuous attention must be paid to the understanding of intrinsic material characteristics and printing process, especially in the aspects of composition regulation, crystallization control, solvent optimization, and interface regulation. In addition, innovations in packaging materials and technology are also critical for significantly extending the lifespan of PSCs.

Third, developing large‐area screen‐printed PSC modules. The main technical challenge faced by large‐area PSCs is the amplification preparation of uniform and high crystallinity perovskite films. Although the fabrication of screen‐printed perovskite film on large‐area substrate has been realized, the film quality is still needed to be improved. Therefore, further optimizing the characteristics of perovskite printing ink and the crystallization process of perovskite can effectively improve the quality of perovskite films and the performance of modules. In addition, cost‐reduction is also an important challenge for perovskite photovoltage modules. Owing to the patterning‐applicable advantage of screen‐printing method, it can simplify the preparation process and reduce electricity consumption by eliminating the laser etching process Therefore, the integrated and reliable fully screen‐printed PSCs technology proposed in this work combined with roll‐to‐roll process is expected to further reduce the cost of PSC modules and improve the competitiveness of perovskite photovoltage products.

## Conflict of Interest

The authors declare no conflict of interest.
